# Comparative genomics analysis of statistically significant genomic islands of *Helicobacter pylori* strains for better understanding the disease prognosis

**DOI:** 10.1042/BSR20212084

**Published:** 2022-03-16

**Authors:** Joyeeta Chakraborty, Raghunath Chatterjee

**Affiliations:** Human Genetics Unit, Indian Statistical Institute, 203 B T Road, Kolkata 700108, India

**Keywords:** Genomic Island, Horizontal Gene Transfer, Pathogenicity Island, Toxin-antitoxin system

## Abstract

Bacterial virulence factors are often located in their genomic islands (GIs). *Helicobacter pylori*, a highly diverse organism is reported to be associated with several gastrointestinal diseases like, gastritis, gastric cancer (GC), peptic ulcer, duodenal ulcer (DU) etc. A novel similarity score (*Sm*)-based comparative analysis with GIs of 50 *H. pylori* strains revealed clear idea of the various factors which promote disease progression. Two putative pathogenic GIs in some of the *H. pylori* strains were identified. One GI, having a putative labile enterotoxin and other dynamin-like proteins (DLPs), is predicted to increase the release of toxin by membrane vesicular formation. Another island contains a virulence-associated protein D (*vapD*) which is a component of a type-II toxin–antitoxin system (TAs), leads to enhance the severity of the *H. pylori* infection. Besides the well-known virulence factors like Cytotoxin-associated gene A (*CagA*) and *vacA*, several GIs have been identified which showed to have direct or indirect impact on *H. pylori* clinical outcomes. One such GI, containing lipopolysaccharide (LPS) biosynthesis genes was revealed to be directly connected with disease development by inhibiting the immune response. Another collagenase-containing GI worsens ulcers by slowing down the healing process. GI consisted of *fliD* operon was found to be connected to flagellar assembly and biofilm production. By residing in biofilms, bacteria can avoid antibiotic therapy, resulting in chronic infection. Along with well-studied *CagA* and vacuolating toxin A (*vacA*) virulent genes, it is equally important to study these identified virulence factors for better understanding *H. pylori*-induced disease prognosis.

## Introduction

*Helicobacter pylori*, formerly known as *Campylobacter pylori*, is a Gram-negative, microaerophilic bacteria that is part of the gastric microbiota in over 50% of population worldwide. The International Agency for Research on Cancer (IARC) classified it as a Class I carcinogen in 1994, nearly 20 years after its discovery, stating that people having *H. pylori* infection have six-times higher risk of developing gastric cancer (GC) than those who do not [1]. Colonization of *H. pylori* in stomach is commonly acquired in early age, mostly by person-to-person transmission through fluidic or aerosolic transfer mediated by the feco-oral route [2,3]. *H. pylori* infection leads to a variety of upper gastrointestinal disorders, such as chronic gastritis, peptic ulcer disease (PUD), gastric mucosa-associated lymphoid tissue (MALT) lymphoma, and GC [4–6]. Colonization persists throughout life unless treated with antibiotics. Unfortunately, the spike in antibiotic resistance is clearly affecting the response to the therapy and there is currently no preventive vaccine measure. Different strains of the *H. pylori* were found to have different level of virulence, while some of the strains were found to be non-virulent. So, to understand the disease prognosis in a better way and to develop new therapeutics to fight against this bacterium, it is important to study *H. pylori* at the strain level. Strain-specific virulence factors are generally located in the Genomic Island (GI) region of particular pathogenic strains. Large genomic regions consisting of multiple horizontally transferred genes (HTGs) are collectively termed as GIs [7]. These are crucial for bacterial evolution, adaptability, and diversification as they contain genes that code for a wide range of proteins [8]. In 1990, Hacker et al. identified virulent genes in uropathogenic *strains* of *Escherichia coli*. This identified virulence factor was not present in beneficial strains of *E. coli*. This cluster of genes was referred to as pathogenicity islands (PAIs). It has been proposed that possible vaccine candidates are located within PAIs [9]. Several other reports suggested that apart from carrying virulent factors, GIs can have clusters of genes related to special biological functions such as, genes required for adaptation in a special environment, genes encoding proteins linked to metabolic processes, antimicrobial resistance genes. Based on the function of the genes present on GIs, they can be differentiated as metabolic islands (MIs), resistance islands (REIs) etc. [10,11].

Despite the fact that *H. pylori* infection is a leading cause of GC, the majority of people who carry the bacteria in their stomachs never get diagnosed with GC. It was also found that some of the normal individuals showed to have higher abundance of *H. pylori.* They neither showed any symptoms nor had any discomfort. *H. pylori* appeared to be the part of normal microflora for these samples. So the role of *H. pylori* in GC prognosis is still unclear. *H. pylori* is a highly diverse organism. Studying *H. pylori* at strain level may shed light to understand the disease prognosis. In the present study, the GIs were thoroughly studied in 50 *H. pylori* strains in order to find strain-specific novel virulent factors and other Islands. The comparative study with the GIs among 50 *H. pylori* strains identified GIs with virulence-associated genes in addition to the known GIs. Further characterization of the newly detected islands identified the possible function of these GIs. Several other factors were also identified which were found to be associated either directly or indirectly with the *H. pylori*-induced disease prognosis.

## Methods

### Comparative analysis of GIs in *H. pylori* genomes

Fifty completely sequenced strains of *H. pylori* were downloaded from ftp://ftp.ncbi.nlm.nih.gov/. All of these strains had common hosts i.e. humans. Geographical distribution and their disease association are listed in Supplementary Tables S1 and S2. *Design-Island-II* (https://www.isical.ac.in/∼rchatterjee/Design-Island.htm), a *de novo* algorithm for GI predication was run on 50 *H. pylori* strains to predict the statistically significant GIs. Islands that were ≥10 kb in length were annotated as GIs. All HTGs within these GIs were identified from the gene annotation data using an in-house perl script. For comparison of the GIs among different strains, we used cd-hit suite (http://weizhong-lab.ucsd.edu/cdhit-web-server/) to assign unique Cluster numbers (CLS) to the HTGs. Sequences with 90% identity were given identical cluster (CLS) numbers.

### Similarity score-based visualization tool

To identify the similarity of a particular GI among different *H. pylori* strains, we introduced a similarity score (*Sm*):

$$Sm=\frac{\text{A}\cap \text{B}}{\text{A}\cup \text{B}}$$

where, A and B correspond to the number of genes within the given GIs as identified by *Design-Island-II.* *Sm* calculates the number of common genes present in the two GIs, divided by the number of total genes present in the two GIs. We calculated *Sm* score for all the identified GIs in 50 *H. pylori* strains. *Sm* value*-*based heat maps were generated for all GIs to get an idea about the similarities or dissimilarities among the 50 *H. pylori* strains. Hierarchical clustering on the *Sm* score and heat maps were generated for all GIs using R packages (gplots and heatmap.2).

### Core and accessory island

A cut-off value of *Sm* ≥ 50% was used to annotate a GI as shared between two strains. GIs present in all 50 strains were classified as core GIs. These were named as Core_1, Core_2, etc. Accessory GIs were defined as GIs found in 2–49 *H. pylori* strains and termed as Acc_1, Acc_2, Acc_3 and so on. The presence and absence of GIs across all the strains were denoted as 1 and 0. Kendall’s Tau correlation coefficient was calculated among all 50 *H. pylori* strains considering the presence or absence of accessory islands.

### Annotation of hypothetical proteins

A large number of genes within many accessory islands were annotated as hypothetical proteins. Functional characterization of these unknown proteins was performed using a conserved domain search (https://www.ncbi.nlm.nih.gov/Structure/cdd/wrpsb.cgi) and psi blast (NCBI) against PDB database with default parameters until convergence. The query sequences were searched against CDD v3.19 - 58235 PSSMs database, using 0.010 as expected value. To understand the conservation of the homolog in the organisms and their evolutionary relationships, multiple sequence alignment and phylogenetic analysis were carried out using Molecular Evolutionary Genetic Analysis (MEGA) [12].

### Protein–protein interaction and homology modeling

The probable biological role of the uncharacterized proteins is predicted by studying the protein–protein interaction using STRINGv10 [13]. Three-dimensional protein structures were generated using an automatic fold recognition server Phyre2 [14]. The PDB IDs of the templates used for homology modeling of putative enterotoxin, virulence-associated protein D (vapD) and DUF3240 family protein were 4AUR (Confidence: 99.9%; Coverage: 95%), 3UI3 (Confidence: 100.0%; Coverage: 96%) and 3CE8 (Confidence: 93.5%; Coverage: 75%), respectively. Clashes, rotamers and Ramachandran analysis were done by Molprobity [15]. Fpocket2 and CSA programs were used to detect pocket and catalytic site of the hypothetical protein, respectively [16,17].Visualization and modification of the 3D model were carried out in PyMOL [18].

### Docking

Protein–peptide interactions are essential in various biological processes involving signaling, cellular localization etc. Twenty-six probable complexes were generated using a protein–protein docking server ClusPro [19]. The complex that had the highest binding energy and lowest free energy was considered. In a protein complex some of the residues take part in the interaction. BIPSPI was used to predict partner-specific binding sites in the protein–protein complex [20]

### Analysis of GIs with other GI prediction tools

To validate the identified GIs with other GI prediction tools, sequence composition-based approaches like, PredictBias [21], Zisland Explorer [22], MSGIP [23]; comparative genomics approach like, IslandPick [24]; and hybrid approach like IslandViewer 4 [25] were selected. These methods were applied on 50 *H. pylori* strains to identify the GIs and their locations in the genome. The length of the predicted GIs were compared with that of the GIs predicted by *Design-Island-II*. If at least 50% of the length of GI, predicted by any other tool was overlapped with our predicted GIs, it was determined as detected by both methods.

## Results

### Identification of GIs across 50 *H. pylori* strains

**Figure 1:**
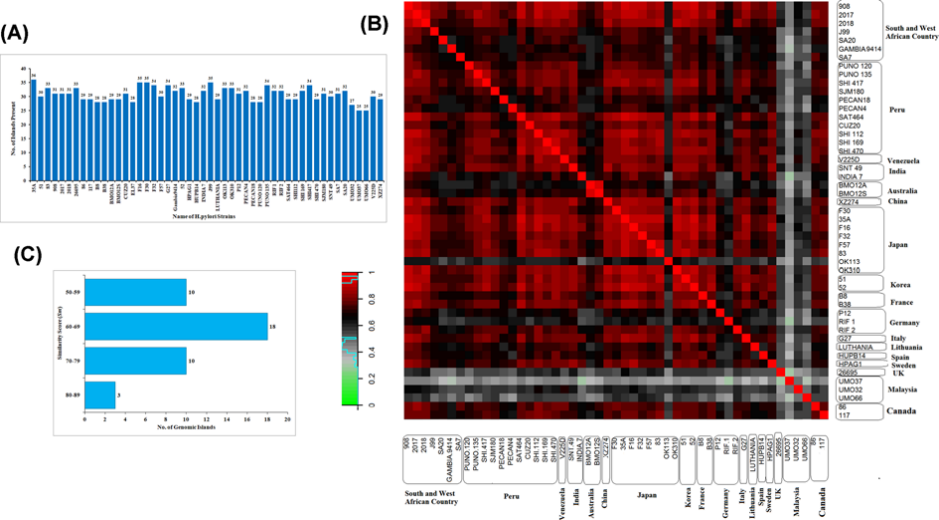
GI distribution in 50 *H. pylori* strains (**A**) Distribution of 41 statistically significant GIs in 50 *H. pylori* strains. (**B**) Heat map with the Kendall Tau’s correlation coefficients considering presence or absence of accessory islands among different *H. pylori* strains. (**C**) Distribution of genomic islands on the basis of *Sm* score.

*Design-Island-II*, a modified version of *Design-Island* [26] was used to detect statistically significant GIs in all 50 *H. pylori* strains. A total of 41 GIs with ≥10 kb length were included for the comparative analysis of 50 strains. The *H. pylori 35A* strain had the highest number of GIs (36 GIs), followed by two Fukui strains (*H. pylori F16* and *H. pylori F30*) and *H. pylori J99*, both of which had 35 GIs. The Malaysian strains *H. pylori UM037* and *H. pylori UM066* had the lowest number of predicted GIs (25 GIs) (Figure 1A). Heat map using Kendall’s Tau correlation coefficient with the GIs among all 50 strains showed clustering of strains according to their place of isolation. The higher degree of correlation among geographically related strains suggested a region-specific interstrain HGT among strains. Like, Australian strains *H. pylori BM012A* and *H. pylori BM012S* shared almost identical GIs distribution between them, but these were not so similar to other strains isolated from other geographical locations. South East Asian strains, specifically the Malaysian strains (*H. pylori UM032, H. pylori UM037* and *H. pylori UM066)* were quite different from each other and other geographical strains. On the contrary, the strains from Japan, Korea and France showed ≥0.80 correlation among themselves. South African strains *H. pylori 908, H. pylori 2017* and *H. pylori 2018* were almost identical in terms of shared GIs (Figure 1B).

### Comparative analysis of GIs in *H. pylori*

Forty-one GIs identified in all 50 strains were subjected to comparative analysis among all *H. pylori* strains. Among these, seven GIs were present in all 50 strains and were termed as core GI. Three core GIs (Core_1 to Core_3) were found to have *Sm ≥* 80%. The rest four core GIs (Core_4 to Core_7) had *Sm ≤* 80%. Thirty-four accessory GIs (namely, Acc_1 to Acc_34) were present in 2–49 *H. pylori* strains with (*Sm* ≤ 80%) (Figure 1C). The plausible functions of all the 41 GIs were predicted (Supplementary Table S3). Two of the seven core GIs were mainly enriched with ribosomal proteins. In terms of composition, many highly expressed genes, like ribosomal proteins, deviate from the genomic background [27,28]. Thus, they were identified as GIs despite the fact that they were part of the host genome. Various studies suggested eliminating ribosomal proteins containing GIs, as these are false positive in terms of identification [29,30].

### Characterization of identified core GIs

#### Antibiotic resistance island

Core_3 was found to be present in all *H. pylori* strains with an average *S_m_* = 83.1%. The heat map gave a lucid pictorial illustration of the similarity of Core_3 GI among strains (Supplementary Figure S1A). The length of Core_3 was ∼17 kb and 14 HTGs were present on it. This gene cluster was the entire nuo operon (NADH: ubiquinone oxidoreductase). This operon contains essential genes with unique genomic properties, such as different oligonucleotide frequencies and slightly greater GC% than tissue-specific genes differentiate this region from rest of the genome [31,32]. Thus, this nuo operon was identified as GI. Benzimidazole (BI) derivatives resistant *H. pylori* strains have three mutations in NADH-quinone oxidoreductase subunit D (NuoD) at G398S, F404S and V407M and one in NADH-quinone oxidoreductase subunit B (NuoB) at T27A. Despite the fact that this GI contains essential genes, it can be classified as an antibiotic-resistant island. The synteny of 14 genes in this GI revealed a framework in which homologous gene conservation and gene order were observed (Supplementary Figure S1B).

#### Vacuolating toxin A PAI

Core_4, a ∼17 kb long GI was detected to contain vacuolating toxin A (*vacA*) gene along with another toxin flippase-type exporter (*mviN*), iron (III) dicitrate ABC transporter genes *fecD* and *fecE*, short-chain oxidoreductase (*vdlC*) in almost all. *VacA* was deleted in five *H. pylori* strains like, *H. pylori Shi169, H. pylori UM037, H. pylori Rif1, H. pylori Rif2, H. pylori SouthAfrica7.* In *H. pylori 52* and *H. pylori BM012S vacA* was annotated as non-coding. Synteny of this GI revealed that it differed between strains depending on gene content (Supplementary Figure S2A) with average *Sm* score of 77%. The hierarchical clustering based on the *Sm* grouped the samples in two major clusters (Supplementary Figure S2B). This GI is associated with virulence as well as iron transport. *H. pylori Rif1, H. pylori Rif2* strains lacked *vacA* gene, though in the other *vacA* lacking strains, *vacA* paralogs were present on another accessory GI, Acc_23.

### Niche adaptation island (urease)

**Figure 2:**
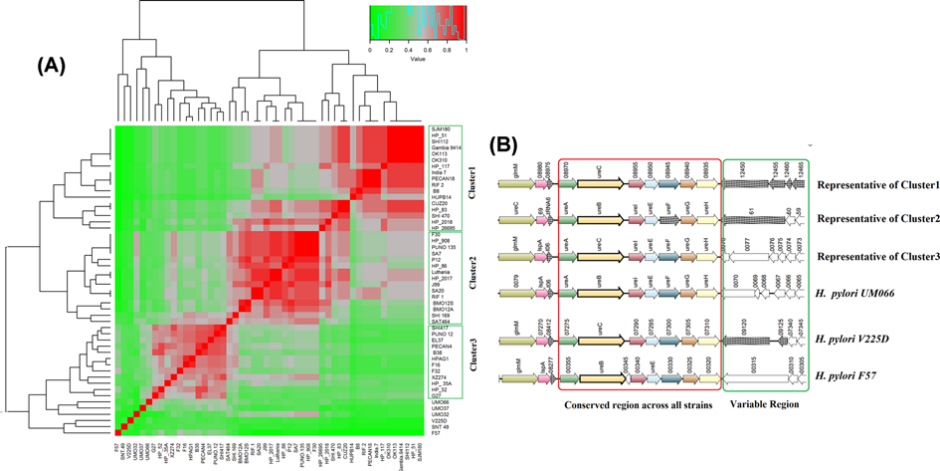
Comparative genomic study of Niche Adaptation Island (Core_5) in 50 strains of *H. pylori* (**A**) Heat map of *Sm* using hierarchical clustering. Red: *S_m_* ≥ 0.70; Gray: *S_m_* ≥ 0.4–0.69, Green: *S_m_* ≤ 0.39. Fifty strains showed three main clusters. (**B**) Synteny of representative strains from each cluster. The region highlighted in red is the most conserved part consists of seven genes in the Urease Island and the variable region is highlighted in green.

The urease gene cluster (Core_5), which was adopted horizontally, became one of the most important genetic elements for the survival of *Helicobacter* strains. The GI was ∼17.5 kb in length and consisted of 16 HTGs. Genes encoding *H. pylori* urease are located as a single 6.13-kb gene cluster that consists of seven contiguous genes; *ureA*, *ureB*, *ureI*, *ureE*, *ureF*, *ureG* and *ureH*, are necessary for the synthesis of an active enzyme [33,34]. The average *Sm* was found to be 58%. The heat map with the *Sm* of Urease Island showed three distinct clusters (Figure 2A). Three major clusters were observed in this GI. The insertion or deletion of genes around the conserved seven genes resulted in three different clusters. There were insertion of hypothetical genes and non-coding sequence in the downstream region of the conserved urease gene cluster. Malaysian strains (*H. pylori UM032, H. pylori UM037, H. pylori UM066*), Venezuela strain *H. pylori V225*, Fukui strain *H. pylori F57* showed distinct gene arrangements (Figure 2B). The effective colonization by the bacteria in the host is the first stage towards conferring pathogenicity.

### PAI containing collagenase

Core_6, a 13-kb long GI, was identified with genes that promote the virulence of *H. pylori* infection. This GI was present in all strains, whether intact, fragmented or missing in part. However, a U32 family peptidase was present in all strains. Other important HTGs of this GI were a DNA-binding response regulator (*racR*), peptide chain release factor RF2 (*prfB*), flagellar biosynthetic protein FliR (*fliR*), fructose-bisphosphate aldolase (*fbaB*), etc. The average *Sm*, calculated on the basis of gene content was found to be 64.5%. Thirty-six strains showed >80% similarity and formed the main cluster in the hierarchical clustering (Supplementary Figure S3A). U32 family peptidase present in all the strains is actually a collagenase. Gastric epithelium extracellular matrix is mostly composed of collagen type I and III. Type I collagen synthesis is increased in the areas surrounding gastric ulcers. Thus, ulcer healing process is promoted [35]. However, the release of collagenase from *H. pylori* inhibits the healing process, thus increase the chronicity of the ulceration. Synteny of this GI showed all the 50 strains have this virulence factor, but the difference in the gene content of the adjacent region led to formation of distinct clusters (Supplementary Figure S3B).

### Niche adaptation (biofilm formation)

Core_7 was found to contain *fliD* operon along with some other HTGs, like, DNA-damage induced multidrug efflux protein (*dinF*), N-carbamoylputrescine amidase (*cpa*), 2′,3′-cyclic-nucleotide 2′-phosphodiesterase (*ymdA*) etc. This GI was ∼12 kb long and was present in all strains. The *fliD* operon not only promotes *H. pylori* colonization in the human stomach by forming flagella, but it also facilitates biofilm formation [36]. The bacteria can evade antibiotic therapy by living in biofilms, leading to chronic infection [37]. This operon was intact across all the 50 *H. pylori* strains; with 75% of average *Sm* score (Supplementary Figure S4A). The difference in adjacent gene cluster led to the formation of two major groups (Supplementary Figure S4B). Core_7 is involved in *H. pylori* colonization and biofilm formation, which contributes to the disease’s chronicity. As *fliD* is present in all strains and crucial for *H. pylori* survival, it can be a potential target for new drug or vaccine development.

### Accessory GIs associated with virulence

#### Lipopolysaccharide biosynthesis island

**Figure 3:**
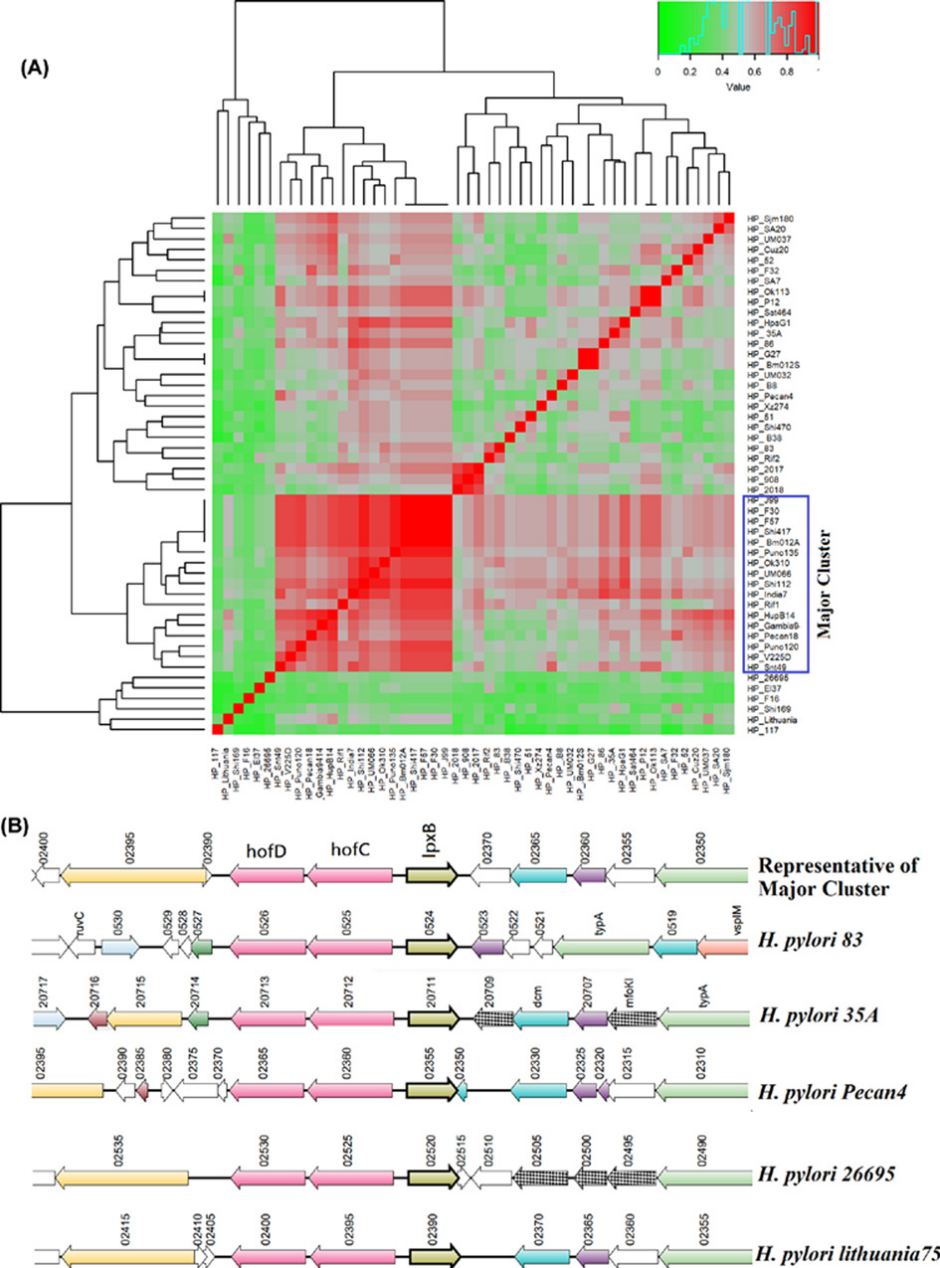
Comparative genomic study of Lipopolysaccharide Biosynthesis Island (Acc_17) in 50 strains of *H. pylori* (**A**) Heat map of *Sm* using hierarchical clustering. Red: *S_m_* ≥ 0.70; Gray: *S_m_* ≥ 0.4–0.69, Green: *S_m_* ≤ 0.39. Fifty strains showed one major cluster. (**B**) Synteny of representative strain from main cluster and other strains.

Acc_17 GI was ∼13 kb in length and contained ten HTGs present on it, like, lipid-A-disaccharide synthase (*lpxB*), hof-family outer membrane protein (*hofC*, *hofD*), CDP-diacylglycerol diphosphatase (*cdh*) etc. This GI was found in 41 strains, with an average *Sm* of 55.3%. The hierarchical clustering in the heat map divided the strains into two major clusters (Figure 3A). Synteny of this GI also represented that the gene cluster was not intact in all strains, and it was fragmented or some genes were missing (Figure 3B). This GI was found to be associated with lipopolysaccharide (LPS) synthesis. Lipid A-core and the O antigen polysaccharide make up LPS which serves as a key component on the surface of *H. pylori*. Lewis antigens are present in the O polysaccharides which imitate the glycan structures produced by human cells. Lewis antigens interact with human dendritic cells, generating an immune response that contributes to *H. pylori* pathogenicity [38]. As LPS is involved in producing *H. pylori* induced virulence, this GI may indirectly influence the pathogenesis of the strain.

#### CagA PAI

Cag is an exhaustively studied PAI in *H. pylori.* Comparative analysis of the cagPAI (Acc_25) identified that ∼80% of the strains have 70–100% of resemblance among them (Supplementary Figure S5A). It was ∼40 kb in length and consisted of 32 genes. Three strains (*H. pylori 2017, 2018* and *908*) showed ∼90% similarity among themselves, however, differed from the other strains. These three strains, reportedly associated with duodenal ulcer (DU), belong to the same geographical location i.e. Africa. While five Cag negative strains *H. pylori Aklavik 86*, *H. pylori Aklavik 117*, *H. pylori B38*, *H. pylori SA7* and *H. pylori SA20* were clustered together. Hierarchical clustering based on the *Sm*, generated from gene content showed three distinct clusters. Forty-five strains Cag positive strains were either associated with a specific disease or isolated from patients. Four Fukui strains (*H. pylori F16, H. pylori F30, H. pylori F32* and *H. pylori F57*), two Australian (*H. pylori BM012A* and *H. pylori BM012S*), two German (*H. pylori Rif1* and *H. pylori Rif2*) and two Korean (*H. pylori 51* and *H. pylori 52*) strains showed to have identical gene content among themselves, suggesting an intraspecies horizontal transfer of CagPAI within a particular geographical location. Synteny of this island showed three representatives from three distinct clusters observed in the heat map (Supplementary Figure S5B). Gene composition analysis of the cagPAI suggested additive or reductive evolution in some of the *H. pylori* strains.

#### Lysine biosynthesis island

Another biosynthetic island (Acc_14) was identified in 33 *H. pylori* strains. The GI was ∼15 kb long and consisted of 11 HTGs. Diaminopimelate decarboxylase (*lysA*), chorismate mutase, amidase (*amiE*), para-aminobenzoate synthase glutamine amidotransferase (*pabB*) were some of the genes linked to the lysine biosynthetic process. The similarity among the strains was *S_m_* = 69%. Hierarchical clustering identified three distinct clusters. The gene content of Acc_14 in *H. pylori 51, H. pylori 83* and *H. pylori Lithuania 75* were quite different from others (Supplementary Figure S6A). The functional relevance of this particular GI came up with an interesting finding. Previous reports suggested consumption of a lot of salt in the diet has been linked to an elevated risk of stomach cancer [39–42]. Cytotoxin-associated gene A (*CagA*), *amiE*, *lysA*, *lgE2*, and other proteins were found to have increased expression in bacteria cultivated in high-salt conditions [43]. So, this led to the conclusion that the strains containing *amiE*, *lysA* enhance the risk of GC in people who take a high-salt diet. The following strains *H. pylori 35A* (HMPREF4655_20533), *51* (KHP_0287), *H. pylori 908* (hp908_0304), *H. pylori 2017* (hp2017_0297), *H. pylori B8* (HPB8_1272), *H. pylori B38* (HELPY_0296), *H. pylori F30* (HPF30_1004), *H. pylori F32* (HPF32_0300), *H. pylori F57* (HPF57_0344), *H. pylori J99* (jhp0275), *H. pylori OK113* (HPOK113_0299), *H. pylori OK310* (HPOK310_0296), *H. pylori P12* (HPP12_0289), *H. pylori SA20* (HPSA20_0322), and *H. pylori V225D* (HPV225_0308) have *lysA* and *amiE*, which thus increase the chance of GC prognosis. However, *lysA* and *amiE* were absent from *H. pylori Cuz20* and *H. pylori Aklavik86*, respectively (Supplementary Figure S6B).

#### Metabolic island

Acc_20 was discovered in 48 *H. pylori* strains, spanning 12.6 kb in length and including 10 HTGs. Two genes found on this GI were primarily linked with the citric acid cycle: 3-oxoacid CoA-transferase A and 3-oxoacid CoA-transferase B (*Sco A* and *B*; known as *SCOT* complex). Other proteins found on it include acetone carboxylase subunit γ (*acxC*), hydantoin utilization protein A (*hyuA*), and acetyl-CoA C-acetyltransferase (*thiA*), short-chain fatty acids transporter (*atoE*), polysaccharide biosynthesis protein (*wlaX*) etc. On the basis of the gene content the average *Sm* was calculated to be 53.8%. All of the studied strains had *scoA*, *scoB* and *acxC* present on Acc_20. Variations in the neighboring gene content led to formation of four separate clusters among the strains (Supplementary Figure S7A,B).

#### Putative labile enterotoxin containing PAI

**Figure 4:**
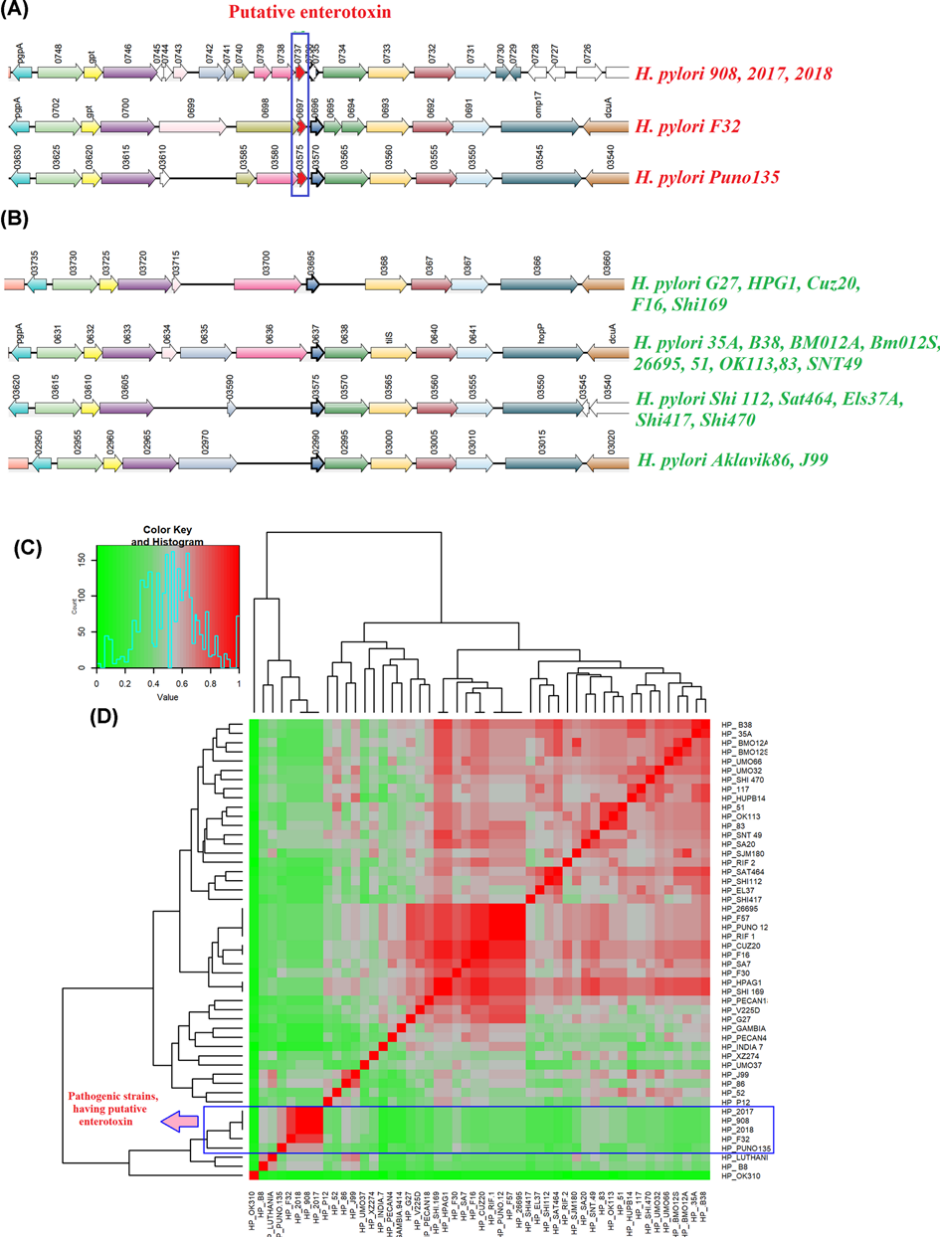
Identification and comparative study of putative toxin in *H. pylori* (**A**) Synteny of five strains showing to have putative enterotoxin. The putative gene highlighted in red is the enterotoxin. (**B**) Synteny of other strains of the same region. (**C**) Heat map of *Sm* using hierarchical clustering. Red: *S_m_* ≥ 0.70; Gray: *S_m_* ≥ 0.4–0.69, Green: *S_m_* ≤ 0.39. Five strains showed similar structural composition. The region highlighted in blue composed of the strains, having putative enterotoxin.

**Figure 5:**
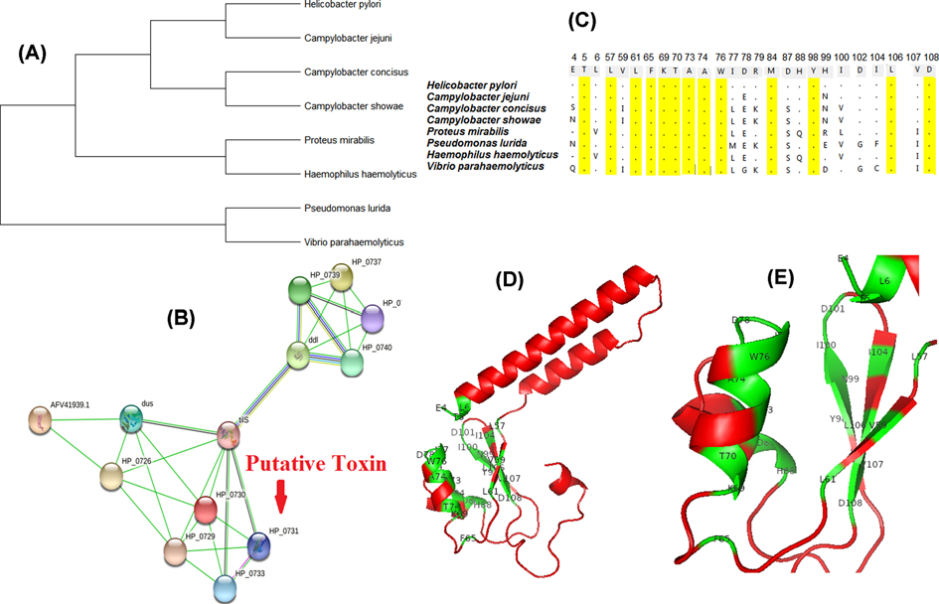
Evolutionary and functional analysis of novel toxin (**A**) Phylogenetic tree of homologs of putative toxin in different bacteria. (**B**) Conservation of residues in the binding site. Yellow highlighted amino acids are conserved in all the studied microorganisms. (**C**). Protein interaction study of putative toxin. Gene neighborhood and gene co-occurence are shown in green and blue colors, respectively. (**D**) Protein 3D homology model of putative toxin. Green highlighted residues are involved in binding pocket formation. (**E**) Zoomed view of the binding pocket.

HTGs of several accessory GIs were annotated as hypothetical proteins. While characterizing the function of these GIs, we identified a GI (Acc_31) consisted of 11 hypothetical proteins with a labile enterotoxin output A. This labile enterotoxin was found only in five pathogenic strains, namely *H. pylori 908, H. pylori 2017* and H. *pylori* 2018, isolated from DU patients. *H. pylori Puno135* and *H. pylori F32* were isolated from gastritis and GC patients, respectively. Synteny study of these strains differed from other *H. pylori* strains (Figure 4A,B), suggesting its role in disease pathogenesis. These five strains were grouped together as having a similarity of ∼95% however; gene content of this cluster was quite dissimilar with other strains (Figure 4C). Apart from the putative labile enterotoxin output A, this island consisted of three dynamin-like protein (DLP) helical superfamily proteins, GTPase, apolipoL family protein, cyclic β-1,2-glucan ABC transporter, RsgAGTPase superfamily and ATP-binding protein. Phylogenetic analysis showed that the closest homolog of this toxin was present in another epsilon bacteria *Campylobacter jejuni.* Other close homologs were found in genera like *Proteus*, *Pseudomonas*, *Haemophilus* and *Vibrio* (Figure 5A). Protein–protein interaction study showed that the putative labile enterotoxin output A was co-expressed and present in the neighborhood of tRNA(*Ile*)-lysidine synthase (*tilS*). *TilS* co-occurred and co-expressed with d-alanine-d-alanine ligase (*ddl*) and tRNA-dihydrouridine synthase (*dus*) (Figure 5B). *Ddl* was associated with peptidoglycan biosynthesis and vancomycin resistance whereas *dus* was associated with tRNA processing. The Protein 3D model of the putative toxin was generated by homology modeling. Ramachandran plot, rotamer study, clash analysis of the built 3D structure gave an idea that it modeled ideally (Supplementary Figure S8A–C).The binding pocket, where it binds with its ligands, was made up of 26 amino acids. Fifty percent residues of this pocket were found to be conserved among various closely related bacteria (Figure 5C). The homology models of the putative toxin and its binding pocket are presented in Figure 5D,E.

#### PAI containing toxin–antitoxin system

**Figure 6:**
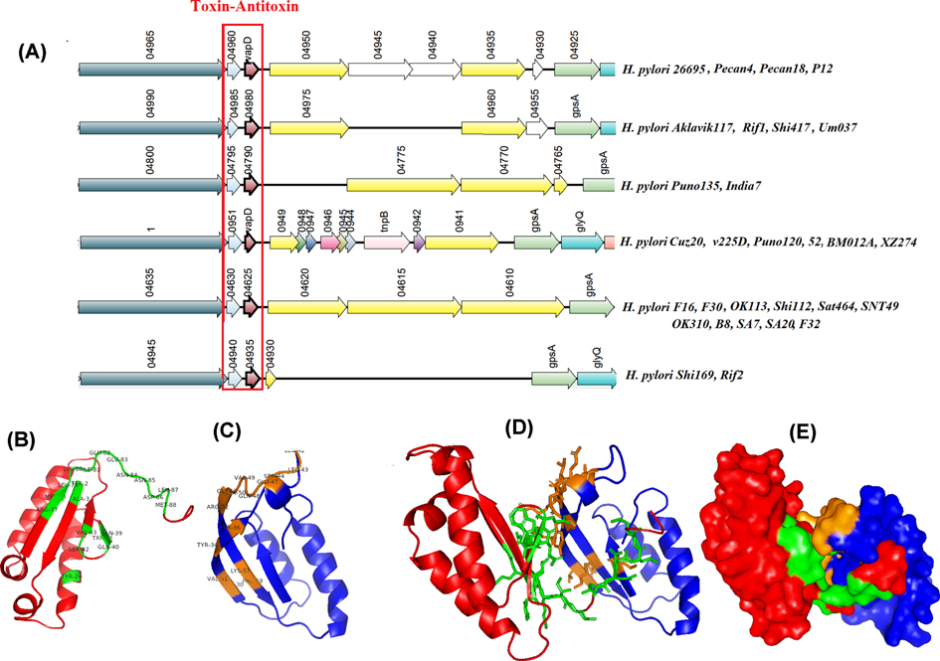
Characterization of TAs (**A**) Synteny study of the representative sequence of the cluster. (**B**) The interacting residues in vapD–antitoxin docked complex. (**C**) Surface structure of the docked protein. (**D**,**E**) Residues responsible for protein–protein interaction between vapD and antitoxin, respectively. Red: vapD, Green: interacting residues of vapD, Blue: DUF3240 family antitoxin, Orange: interacting residues of DUF3240 family antitoxin.

*Design-Island-II* identified a PAI (Acc_33), consisted of a Toxin–Antitoxin system (TAs) in 29 of the studied strains. This GI contains a *vapD*, Cobalt-zinc-cadmium resistance protein, cation system protein, and six proteins with unknown function. These hypothetical proteins were found to be either dynamin or DUF3240 family protein. VapD is a toxin with purine-specific endoribonuclease activity, whereas DUF3240 family protein, with unknown function, binds to its own regulatory region. The toxin and antitoxin were found to be present on the GI of all strains; however, the neighboring genes within the GIs were missing in 26 strains, except *H. pylori* F16, *H. pylori* F30 and *H. pylori* F32 (Figure 6A). In type II TAs, the toxic protein is inhibited post-translationally by binding of a less-stable antitoxin protein. *VapD* and its antitoxin were modeled based on their available homologs. For proper functioning, the toxin–antitoxin components must interact physically through electrostatic forces, hydrogen bonding or hydrophobic effect. To check toxin–antitoxin structural interaction docking is performed. The residues responsible for protein–protein interaction in the docked complex were identified in both vapD and its partner (Figure 6B,C). Among 26 protein complexes; the model having the best interaction energy is presented in Figure 6D,E.

#### Prediction of GIs with other GI prediction tools

*Design-Island-II* detected a total of 34 accessory GIs in fifty strains of *H. pylori*, out of which 23 GIs were also identified by at least one of the other 5 GI prediction tools (Supplementary Table S4). One GI (Acc_25) was predicted by all five methods, while thirteen GIs were predicted by three methods (PredictBias [21], Zisland Explorer [22] and MSGIP [23]), and five GIs were predicted by two, and four GIs were predicted by two GI prediction tools (Supplementary Table S4). Two newly identified putative pathogenic GIs, Acc_31 and Acc_33 were detected by four and three GI prediction tools, respectively. Acc_31 (labile enterotoxin containing PAI) was detected in all five strains by these four methods, while Acc_33 (TAs PAI) were detected in twenty-nine strains by the *Design-Island-II* and MSGIP. PredicBias, on the other hand, identified Acc_31 in twenty-one strains. A synteny analysis revealed that the TAs were present in all strains, but PredictBias was unable to identify this GI, probably because to the lack of surrounding gene content (Supplementary Figure S9).

Eleven accessory GIs were identified only by *Design-Island-II*. Among these, four GIs (Acc_9, Acc_15, Acc_26 and Acc_34) contained ribosomal proteins along with some HTGs of miscellaneous functions. Many highly expressed genes, such as ribosomal proteins, diverge from the genetic background in terms of composition. As a result, despite the fact that they were essential genes, they were identified as GIs. Several studies have proposed that GI-containing ribosomal proteins be eliminated since they are false positives in terms of identification [29,30].

## Discussion

Comparative analysis among 50 strains of *H. pylori* taking led to identify some new statistically significant GIs in addition to the known GIs. Characterization of the detected GIs gave an idea of their impact in the *H. pylori* colonization, infection and disease prognosis. The nuo operon encoding NADH: ubiquinone oxidoreductase or complex I was found as a GI in all 50 strains. Complex I, present in inner mitochondrial membrane, have multisubunits and are responsible for catalyzing the first step of cellular respiration. Essential genes serve as lucrative targets of drug development. The standard *H. pylori* eradication therapy is done using PPIs along with antibiotics like, amoxicillin, metronidazole, clarithromycin. Benzimidazoles (BIs) were found to have potent anti *H. pylori* effect in metronidazole- or clarithromycin-resistant strains. However, three missense mutations at amino acid position G398S, F404S, V407M in *NuoD* and one point mutation at T27A in *NuoB* led to the evolution of BI-resistant strains [44].

The effective colonization by the bacteria in the host is the first stage towards conferring pathogenicity. The urease gene cluster is necessary not only for establishing colonization but also for maintaining infection of *H. pylori*. This multimeric enzyme, made up of 12 *UreA* and *UreB* heterodimers, catalyzes the hydrolysis of urea to create CO_2_ and NH_3_. It serves as a buffer against the acidity of the surrounding environment [45]. CO_2_ and NH_3_ diffuse to the periplasmic region where α-carbonic anhydrase converts CO_2_ into the bicarbonate (HCO_3_^−^) and H^+^. The HCO_3_^−^ maintains the periplasmic pH close to 6.1, assisting *H. pylori* to avoid acidic pH [46]. The H^+^ ions, responsible for imparting acidity is also taken up by NH_3_ in the periplasmic region, another way of acid acclimation [47]. As a result, *H. pylori* combats the deadly effect of the acidic pH. CagPAI is a well-studied virulence factor in *H. pylori* [48]. The presence of the cagA gene, though not a virulence determinant, makes a strain more virulent by eliciting the production of interleukin (IL)-8 by gastric epithelial cells [49]. CagPAI encodes *cagA* an onco-protein which gets transferred to the epithelial cells through T4SS. *VacA* is known to form vacuole, facilitates *H. pylori* colonize and a pore-forming toxin. The recruitment of inflammatory cells by *VacA* promotes ulceration. *VacA* mutant strains were found to produce stomach ulcers less likey than wildtype strains in gerbils [50]. CagA, and VacA gene containing GIs have direct impact on GC prognosis, after successful colonization of *H. pylori* in the human stomach mucosa with the help of urease gene cluster containing GI. Thus these three GIs play significant role in the development of *H. pylori* infection-mediated illnesses.

After the successful colonization *H. pylori* damages the insulating mucosal barrier of the stomach and duodenum, allowing acid to penetrate the delicate lining beneath. The acid and germs both damage the lining, resulting in a sore or ulcer. The rapid regeneration of the epithelium layer is not the only mechanism for healing injury; fibrillar collagens of types I and III have been reported to be overexpressed [35]. These simultaneous healing processes are found in gastric or duodenal ulcer. Core_6 was found to contain a U32 family protein, which is a collagenase in nature. This protein degrades the collagen found in the extracellular matrix of the stomach mucosa, as well as the collagen involved in ulcer recovery. By delaying the healing process, it thus increases the severity of ulcer. Thus, Core_6 was found to be associated with promoting ulceration.

Acc_20 was found to contain SCOT complex associated with citric acid cycle and *acxC*, a subunit of *acxABC* responsible for acetone carboxylation. While *H. pylori* lacks succinate dehydrogenase, a key enzyme in Krebs cycle, the SCOT complex acts as an alternative for energy metabolism. On the other hand *acxABC* promotes acetone utilization. Thus, these two are beneficial for *H. pylori* survival. Mutation is *acxABC* led to reduce the colonization significantly in the stomach of mice [51]. So, Acc_20 plays an important role by providing an alternative path for energy production and assisting *H. pylori* to colonize in gastric mucosa.

LPS biosynthesis genes such *hofC*, *hofD* and *lpxB* were found to be present as an accessory island. LPS is present in the cellular membrane of all Gram-negative bacteria and plays a crucial role in pathogenesis. LPS has three distinct parts, a core composed of oligosaccharide, lipid A and O antigen. Lipid A is able to trigger fatal immune reaction even in very low concentration, thus known as endotoxin [52]. The core part is found to be conserved in closely related bacteria. The O antigen representing the outer most part of LPS, imitates carbohydrate (glycan) structure found on human epithelial cells by incorporating Lewis antigens on it [53]. The Lewis antigens (Le^x^ and Le^y^) are capable of interacting with DC-SIGN. It is a type II C-type lectin present on the dendritic cells of gastric epithelium. This interaction signal down-regulates the inflammatory cascade [54]. This suppression of the immune response facilitates a chronic *H. pylori* infection. Thus this GI is also associated with the disease prognosis indirectly. GC is a multifactorial disease which includes genetic and environmental factors. The high salt in-taking diet habit was found to be linked with increase the virulence of *H. pylori*. The virulence *cagA* positive strains were found to be enhanced with heavily salted diet in mice. It elevated the *H. pylori* colonization in the gastric mucosa and led to greater depletion of parietal cell in mice, thus facilitating the development of GC [41]. This result was observed when similar experiment was done with *cagA* negative strains. So, rich salt diet promotes the GC prognosis in *H. pylori* infected people. Acc_14 was discovered to have a number of genes whose overexpression has been linked to a high-salt diet, for example, *amiE*, *lysA*. The exact function of these proteins in GC prognosis not yet understood.

The colonization potential of *H. pylori* is critically influenced by flagellar motility. The *fliD* mutant strain was completely non-motile, was unable to colonize the stomach mucosa of host mice [55]. The *fliD* operon is linked to flagellar assembly and biofilm formation was found on Core_7. Many bacteria employ flagella to assist swarming, adhesion and biofilm formation. In biofilms, *H. pylori* may be able to withstand environmental challenges such stomach acidity and ROS produced by phagocytic cells. The bacteria can evade antibiotic therapy by living in a biofilms, leading to chronic infection [37]. Flagellar hook-associated protein 2 (*fliD*) was found to be present across all the *H. pylori* strains and associated with essential function like flagellar biosynthesis and biofilm formation. It can be considered as a good target for development of a new therapeutic to combat with the *H. pylori* infection.

Characterization of accessory GI (Acc_31) led to the identification of a putative labile enterotoxin. Phylogenetic analysis suggested that this toxin was horizontally acquired from another helical-shaped, Gram-negative bacterium *C. jejuni*, which is the most common cause of food poisoning in Europe and the United States [56]. Further analysis showed that this toxin interacts with ligase *tilS*, an essential protein for viability. *TilS* along with *dus* work in tRNA processing. Apart from this, *tilS* co-expresses with *ddl* which is involved in the peptidoglycan biosynthesis pathway. Thus, putative toxin–*tilS* interaction indirectly helps in tRNA processing, cell wall biosynthesis and vancomycin resistance. Generally, enterotoxins showed a marked effect in the gastrointestinal tract by increasing the chloride permeability of the intestinal mucosal cells [57–60]. The unique ligand of the putative toxin was sulfate ion, known to inhibit electrolyte absorption and increased intestinal motility. These two are the key regulators of food poisoning. Putative labile enterotoxin output A, a GTPase virulence factor, a DLP enhances toxin release, potentially through vesicle secretion [61]. The cellular location of this putative toxin is in the periplasmic region. We can vouch the novel toxin along with other DLPs present on the GI involve in vesicle formation, membrane fusion, thus lead to increase toxin release.

Toxin–antitoxin genes are often inherited through HGT [62]. Three Fukui strains (*H. pylori F30, H. pylori F16 and H. pylori F32*) contain type II TAs which belong to Vap family and encoded by bicistronic operon. *VapD* was present in 60% of the *H. pylori* genomes [63]. *Design-Island-II* identified these TAs in 29 strains. The lengths of the GI in 26 strains were <10 kb, due to deletion of neighboring gene content. Therefore, we studied this GI only in the aforementioned strains. Docking of vapD and its antitoxin forms a stable complex with minimum free energy. The antitoxin binds to *vapD* in the post-translational stage and inhibits its expression. The exact biological role of the *vapD* protein is yet to be established. Several reports suggest VapD in *Haemophilus influenzae*, acts as a toxin and in *Rhodococcus equi* helps in survival through acid tolerance [64,65]. In adenocarcinoma gastric cells and gastric biopsies, vapD–antitoxin expression values were higher than cagA and vacA cytotoxin genes. Another report showed treatment of biopsy samples with chloramphenicol and kanamycin induced the expression of TAs. These suggested that vapD is a virulent factor, protects the bacterium by aiding biofilm formation [66,67]. It helps the bacterium to survive in a hostile environment. Thus, the presence of *H. pylori* in the gastric mucosa for a long time leads to the formation of severe lesions. Integrating all the reports we can presume, under stress and an unfavorable environment the partner antitoxin represses its own regulation, thus aiding the virulence of *vapD*.

Various other GI prediction tools were used to validate the identified GIs by the *Design-Island-II.* This method is an unsupervised method, uses Monte-Carlo statistical test on randomly selected segments of a chromosome. It calculates the oligonucleotide frequencies and performs a statistical test to identify the GIs. Other sequence composition-based approaches, like PredictBias and MSGIP showed comparable prediction with *Design-Island-II.* In contrary, comparative genomics approach (IslandPick) and hybrid approach (IslandViewer 4) predicted GIs were somewhat different from *Design-Island-II* predicted results. However, experimental validation of these predicted GIs and their functional implication in disease pathogenesis need to be explorted to get a better insight on the role of these GIs.

Finally, we can conclude, along with well-studied* cagA* and *vacA* virulent genes, it is equally important to study other virulence factors, as these have direct or indirect impact in the chronicity of gastric diseases like, some of them were linked to induce ulceration or bacterial colonization, while others aided in biofilm development, allowing bacteria to elude therapy and promote persistent infection.

## Supplementary Material

Supplementary Figures S1-S9 and Tables S1-S4

## Data Availability

The data generated in the manuscript are provided in supplementary files. The sources of data used in this manuscript are referred in the manuscript.

## Abbreviations

*cagA*: cytotoxin-associated gene A
DLP: dynamin-like protein
DU: duodenal ulcer
GC: gastric cancer
GI: genomic islands
HTG: horizontally transferred gene
LPS: lipopolysaccharide
NuoB: NADH-quinone oxidoreductase subunit B
NuoD: NADH-quinone oxidoreductase subunit D
PAI: pathogenicity islands
Sm: similarity score
TAs: toxin–antitoxin system
*vacA*: vacuolating toxin A
*vapD*: virulence-associated protein D

## References

[1] Conteduca V. et al. (2013) *H. pylori* infection and gastric cancer: state of the art (review). Int. J. Oncol. 42, 5–18 10.3892/ijo.2012.1701 23165522

[2] Rolig A.S. et al. (2012) Helicobacter pylori requires TlpD-driven chemotaxis to proliferate in the antrum. Infect. Immun. 80, 3713–3720 10.1128/IAI.00407-12 22802346 PMC3457577

[3] Fock K.M., Graham D.Y. and Malfertheiner P. (2013) Helicobacter pylori research: historical insights and future directions. Nat. Rev. Gastroenterol. Hepatol. 10, 495–500 10.1038/nrgastro.2013.96 23752823 PMC3973742

[4] Watanabe T. et al. (1998) Helicobacter pylori infection induces gastric cancer in mongolian gerbils. Gastroenterology 115, 642–648 10.1016/S0016-5085(98)70143-X 9721161

[5] Covacci A. et al. (1999) Helicobacter pylori virulence and genetic geography. Science 284, 1328–1333 10.1126/science.284.5418.1328 10334982

[6] Uemura N. et al. (2001) Helicobacter pylori infection and the development of gastric cancer. N. Engl. J. Med. 345, 784–789 10.1056/NEJMoa001999 11556297

[7] Weinstock G.M. (2000) Genomics and bacterial pathogenesis. Emerg. Infect. Dis. 6, 496–504 10.3201/eid0605.000509 10998381 PMC2627964

[8] Juhas M. et al. (2009) Genomic islands: tools of bacterial horizontal gene transfer and evolution. FEMS Microbiol. Rev. 33, 376–393 10.1111/j.1574-6976.2008.00136.x 19178566 PMC2704930

[9] Moriel D.G. et al. (2010) Identification of protective and broadly conserved vaccine antigens from the genome of extraintestinal pathogenic Escherichia coli. Proc. Natl. Acad. Sci. U.S.A. 107, 9072–9077 10.1073/pnas.0915077107 20439758 PMC2889118

[10] Dobrindt U. et al. (2004) Genomic islands in pathogenic and environmental microorganisms. Nat. Rev. Microbiol. 2, 414–424 10.1038/nrmicro884 15100694

[11] Hudson C.M. et al. (2014) Resistance determinants and mobile genetic elements of an NDM-1-encoding Klebsiella pneumoniae strain. PLoS ONE 9, e99209 10.1371/journal.pone.0099209 24905728 PMC4048246

[12] Kumar S. et al. (2008) MEGA: a biologist-centric software for evolutionary analysis of DNA and protein sequences. Brief. Bioinform. 9, 299–306 10.1093/bib/bbn017 18417537 PMC2562624

[13] Szklarczyk D. et al. (2015) STRING v10: protein-protein interaction networks, integrated over the tree of life. Nucleic Acids Res. 43, D447–D452 10.1093/nar/gku1003 25352553 PMC4383874

[14] Kelley L.A. et al. (2015) The Phyre2 web portal for protein modeling, prediction and analysis. Nat. Protoc. 10, 845–858 10.1038/nprot.2015.053 25950237 PMC5298202

[15] Chen V.B. et al. (2010) MolProbity: all-atom structure validation for macromolecular crystallography. Acta Crystallogr. D Biol. Crystallogr. 66, 12–21 10.1107/S0907444909042073 20057044 PMC2803126

[16] Schmidtke P. et al. (2010) fpocket: online tools for protein ensemble pocket detection and tracking. Nucleic Acids Res. 38, W582–W589 10.1093/nar/gkq383 20478829 PMC2896101

[17] Porter C.T., Bartlett G.J. and Thornton J.M. (2004) The Catalytic Site Atlas: a resource of catalytic sites and residues identified in enzymes using structural data. Nucleic Acids Res. 32, D129–D133 10.1093/nar/gkh028 14681376 PMC308762

[18] DeLano W.L. (2002) Pymol: an open-source molecular graphics tool. CCP4 Newsletter on protein crystallography 40, 82–92

[19] Kozakov D. et al. (2017) The ClusPro web server for protein-protein docking. Nat. Protoc. 12, 255–278 10.1038/nprot.2016.169 28079879 PMC5540229

[20] Sanchez-Garcia R. et al. (2019) BIPSPI: a method for the prediction of partner-specific protein-protein interfaces. Bioinformatics 35, 470–477 10.1093/bioinformatics/bty647 30020406 PMC6361243

[21] Pundhir S., Vijayvargiya H. and Kumar A. (2008) PredictBias: a server for the identification of genomic and pathogenicity islands in prokaryotes. In Silico Biol. 8, 223–234 19032158

[22] Wei W. et al. (2017) Zisland Explorer: detect genomic islands by combining homogeneity and heterogeneity properties. Brief. Bioinform. 18, 357–366 26992782 10.1093/bib/bbw019 PMC5429010

[23] de Brito D.M. et al. (2016) A novel method to predict genomic islands based on mean shift clustering algorithm. PLoS ONE 11, e0146352 10.1371/journal.pone.0146352 26731657 PMC4711805

[24] Langille M.G., Hsiao W.W. and Brinkman F.S. (2008) Evaluation of genomic island predictors using a comparative genomics approach. BMC Bioinformatics 9, 329 10.1186/1471-2105-9-329 18680607 PMC2518932

[25] Bertelli C. et al. (2017) IslandViewer 4: expanded prediction of genomic islands for larger-scale datasets. Nucleic Acids Res. 45, W30–W35 10.1093/nar/gkx343 28472413 PMC5570257

[26] Chatterjee R., Chaudhuri K. and Chaudhuri P. (2008) On detection and assessment of statistical significance of genomic islands. BMC Genomics 9, 150 10.1186/1471-2164-9-150 18380895 PMC2362129

[27] Nomura M. (1999) Engineering of bacterial ribosomes: replacement of all seven Escherichia coli rRNA operons by a single plasmid-encoded operon. Proc. Natl. Acad. Sci. U.S.A. 96, 1820–1822 10.1073/pnas.96.5.1820 10051551 PMC33526

[28] Yap W.H., Zhang Z. and Wang Y. (1999) Distinct types of rRNA operons exist in the genome of the actinomycete Thermomonospora chromogena and evidence for horizontal transfer of an entire rRNA operon. J. Bacteriol. 181, 5201–5209 10.1128/JB.181.17.5201-5209.1999 10464188 PMC94023

[29] Tsirigos A. and Rigoutsos I. (2005) A sensitive, support-vector-machine method for the detection of horizontal gene transfers in viral, archaeal and bacterial genomes. Nucleic Acids Res. 33, 3699–3707 10.1093/nar/gki660 16006619 PMC1174904

[30] Garcia-Vallve S. et al. (2003) HGT-DB: a database of putative horizontally transferred genes in prokaryotic complete genomes. Nucleic Acids Res. 31, 187–189 10.1093/nar/gkg004 12519978 PMC165451

[31] Vinogradov A.E. (2003) Isochores and tissue-specificity. Nucleic Acids Res. 31, 5212–5220 10.1093/nar/gkg699 12930973 PMC212799

[32] Wei K., Zhang T. and Ma L. (2018) Divergent and convergent evolution of housekeeping genes in human-pig lineage. PeerJ 6, e4840 10.7717/peerj.4840 29844985 PMC5971102

[33] Labigne A. (1991) Shuttle cloning and nucleotide sequences of Helicobacter pylori genes responsible for urease activity. J Bacteriol. 173, 1920–31 10.1128/jb.173.6.1920-1931.1991 2001995 PMC207722

[34] Cussac V. (1992) Expression of Helicobacter pylori urease genes in Escherichia coil grown under nitrogen-limiting conditions. J Bacteriol. 174, 2466–73 10.1128/jb.174.8.2466-2473.1992 1313413 PMC205883

[35] Gillessen A. et al. (1995) Evidence of de novo collagen synthesis in healing human gastric ulcers. Scand. J. Gastroenterol. 30, 515–518 10.3109/00365529509089782 7569756

[36] Ratthawongjirakul P. (2016) The impacts of a fliD mutation on the biofilm formation of Helicobacter pylori. Asian Pac. J. Trop. Biomed. 6, 1008–1014 10.1016/j.apjtb.2016.10.005

[37] Garcia A. et al. (2014) Biofilm and Helicobacter pylori: from environment to human host. World J. Gastroenterol. 20, 5632–5638 10.3748/wjg.v20.i19.5632 24914322 PMC4024771

[38] Hug I. et al. (2010) Helicobacter pylori lipopolysaccharide is synthesized via a novel pathway with an evolutionary connection to protein N-glycosylation. PLoS Pathog. 6, e1000819 10.1371/journal.ppat.1000819 20333251 PMC2841628

[39] Tsugane S. (2005) Salt, salted food intake, and risk of gastric cancer: epidemiologic evidence. Cancer Sci. 96, 1–6 10.1111/j.1349-7006.2005.00006.x 15649247 PMC11158463

[40] Fox J.G. et al. (2003) Helicobacter pylori-associated gastric cancer in INS-GAS mice is gender specific. Cancer Res. 63, 942–950 12615707

[41] Fox J.G. et al. (1999) High-salt diet induces gastric epithelial hyperplasia and parietal cell loss, and enhances Helicobacter pylori colonization in C57BL/6 mice. Cancer Res. 59, 4823–4828 10519391

[42] Cover T.L. and Peek R.M. Jr (2013) Diet, microbial virulence, and Helicobacter pylori-induced gastric cancer. Gut Microbes. 4, 482–493 10.4161/gmic.26262 23989802 PMC3928160

[43] Voss B.J. et al. (2015) Alteration of the Helicobacter pylori membrane proteome in response to changes in environmental salt concentration. Proteomics Clin. Appl. 9, 1021–1034 10.1002/prca.201400176 26109032 PMC4690801

[44] Mills S.D., Yang W. and MacCormack K. (2004) Molecular characterization of benzimidazole resistance in Helicobacter pylori. Antimicrob. Agents Chemother. 48, 2524–2530 10.1128/AAC.48.7.2524-2530.2004 15215104 PMC434220

[45] Debowski A.W. et al. (2017) Helicobacter pylori gene silencing in vivo demonstrates urease is essential for chronic infection. PLoS Pathog. 13, e1006464 10.1371/journal.ppat.1006464 28644872 PMC5500380

[46] Athmann C. et al. (2000) Local pH elevation mediated by the intrabacterial urease of Helicobacter pylori cocultured with gastric cells. J. Clin. Invest. 106, 339–347 10.1172/JCI9351 10930437 PMC314326

[47] Weeks D.L. et al. (2000) A H+-gated urea channel: the link between Helicobacter pylori urease and gastric colonization. Science 287, 482–485 10.1126/science.287.5452.482 10642549

[48] Censini S. et al. (1996) Cag, a pathogenicity island of Helicobacter pylori, encodes type I-specific and disease-associated virulence factors. Proc. Natl. Acad. Sci. U.S.A. 93, 14648–14653 10.1073/pnas.93.25.14648 8962108 PMC26189

[49] Wiedemann T. et al. (2009) Helicobacter pylori cag-Pathogenicity island-dependent early immunological response triggers later precancerous gastric changes in Mongolian gerbils. PLoS ONE 4, e4754 10.1371/journal.pone.0004754 19270747 PMC2650263

[50] Ogura K. et al. (2000) Virulence factors of Helicobacter pylori responsible for gastric diseases in Mongolian gerbil. J. Exp. Med. 192, 1601–1610 10.1084/jem.192.11.1601 11104802 PMC2193104

[51] Brahmachary P. et al. (2008) The human gastric pathogen Helicobacter pylori has a potential acetone carboxylase that enhances its ability to colonize mice. BMC Microbiol. 8, 14 10.1186/1471-2180-8-14 18215283 PMC2244623

[52] Raetz C.R. and Whitfield C. (2002) Lipopolysaccharide endotoxins. Annu. Rev. Biochem. 71, 635–700 10.1146/annurev.biochem.71.110601.135414 12045108 PMC2569852

[53] Moran A.P. (2008) Relevance of fucosylation and Lewis antigen expression in the bacterial gastroduodenal pathogen Helicobacter pylori. Carbohydr. Res. 343, 1952–1965 10.1016/j.carres.2007.12.012 18279843

[54] Bergman M.P. et al. (2004) Helicobacter pylori modulates the T helper cell 1/T helper cell 2 balance through phase-variable interaction between lipopolysaccharide and DC-SIGN. J. Exp. Med. 200, 979–990 10.1084/jem.20041061 15492123 PMC2211851

[55] Kim J.S. et al. (1999) Molecular cloning and characterization of the Helicobacter pylori fliD gene, an essential factor in flagellar structure and motility. J. Bacteriol. 181, 6969–6976 10.1128/JB.181.22.6969-6976.1999 10559162 PMC94171

[56] Silva J. et al. (2011) Campylobacter spp. as a foodborne pathogen: a review. Front. Microbiol. 2, 200 10.3389/fmicb.2011.00200 21991264 PMC3180643

[57] Lucas M.L. (2005) Amendments to the theory underlying Ussing chamber data of chloride ion secretion after bacterial enterotoxin exposure. J. Theor. Biol. 234, 21–37 10.1016/j.jtbi.2004.11.005 15721033

[58] Lucas M.L. (2008) Enterocyte chloride and water secretion into the small intestine after enterotoxin challenge: unifying hypothesis or intellectual dead end? J. Physiol. Biochem. 64, 69–88 10.1007/BF03168236 18663997

[59] Pinchuk I.V., Beswick E.J. and Reyes V.E. (2010) Staphylococcal enterotoxins. Toxins (Basel) 2, 2177–2197 10.3390/toxins2082177 22069679 PMC3153290

[60] Principato M. and Qian B.F. (2014) Staphylococcal enterotoxins in the etiopathogenesis of mucosal autoimmunity within the gastrointestinal tract. Toxins (Basel) 6, 1471–1489 10.3390/toxins6051471 24776983 PMC4052247

[61] Michie K.A. et al. (2014) LeoA, B and C from enterotoxigenic Escherichia coli (ETEC) are bacterial dynamins. PLoS ONE 9, e107211 10.1371/journal.pone.0107211 25203511 PMC4159319

[62] Ramisetty B.C. et al. (2016) What is the link between stringent response, endoribonuclease encoding Type II toxin-antitoxin systems and persistence? Front. Microbiol. 7, 1882 10.3389/fmicb.2016.01882 27933045 PMC5120126

[63] Cao P. and Cover T.L. (1997) High-level genetic diversity in the vapD chromosomal region of Helicobacter pylori. J. Bacteriol. 179, 2852–2856 10.1128/jb.179.9.2852-2856.1997 9139899 PMC179045

[64] Benoit S. et al. (2001) Induction of vap genes encoded by the virulence plasmid of Rhodococcus equi during acid tolerance response. Res. Microbiol. 152, 439–449 10.1016/S0923-2508(01)01217-7 11446512

[65] Daines D.A., Jarisch J. and Smith A.L. (2004) Identification and characterization of a nontypeable Haemophilus influenzae putative toxin-antitoxin locus. BMC Microbiol. 4, 30 10.1186/1471-2180-4-30 15274747 PMC503385

[66] Cardenas-Mondragon M.G. et al. (2016) Transcriptional profiling of type II toxin-antitoxin genes of Helicobacter pylori under different environmental conditions: identification of HP0967-HP0968 system. Front. Microbiol. 7, 1872 10.3389/fmicb.2016.01872 27920769 PMC5118875

[67] Morales-Espinosa R. et al. (2020) High expression of Helicobacter pylori VapD in both the intracellular environment and biopsies from gastric patients with severity. PLoS ONE 15, e0230220 10.1371/journal.pone.0230220 32163505 PMC7067408

