# Supplementary material for: Comparative genomics analysis of statistically significant genomic islands of Helicobacter pylori strains for better understanding the disease prognosis
Source: Biosci Rep. 2022 Mar 16;42(3):BSR20212084. doi: 10.1042/BSR20212084 (PMC8935386; doi:10.1042/BSR20212084)
Supplement: Supplementary Figures S1-S9 and Tables S1-S4 [file BSR-2021-2084_supp.pdf]

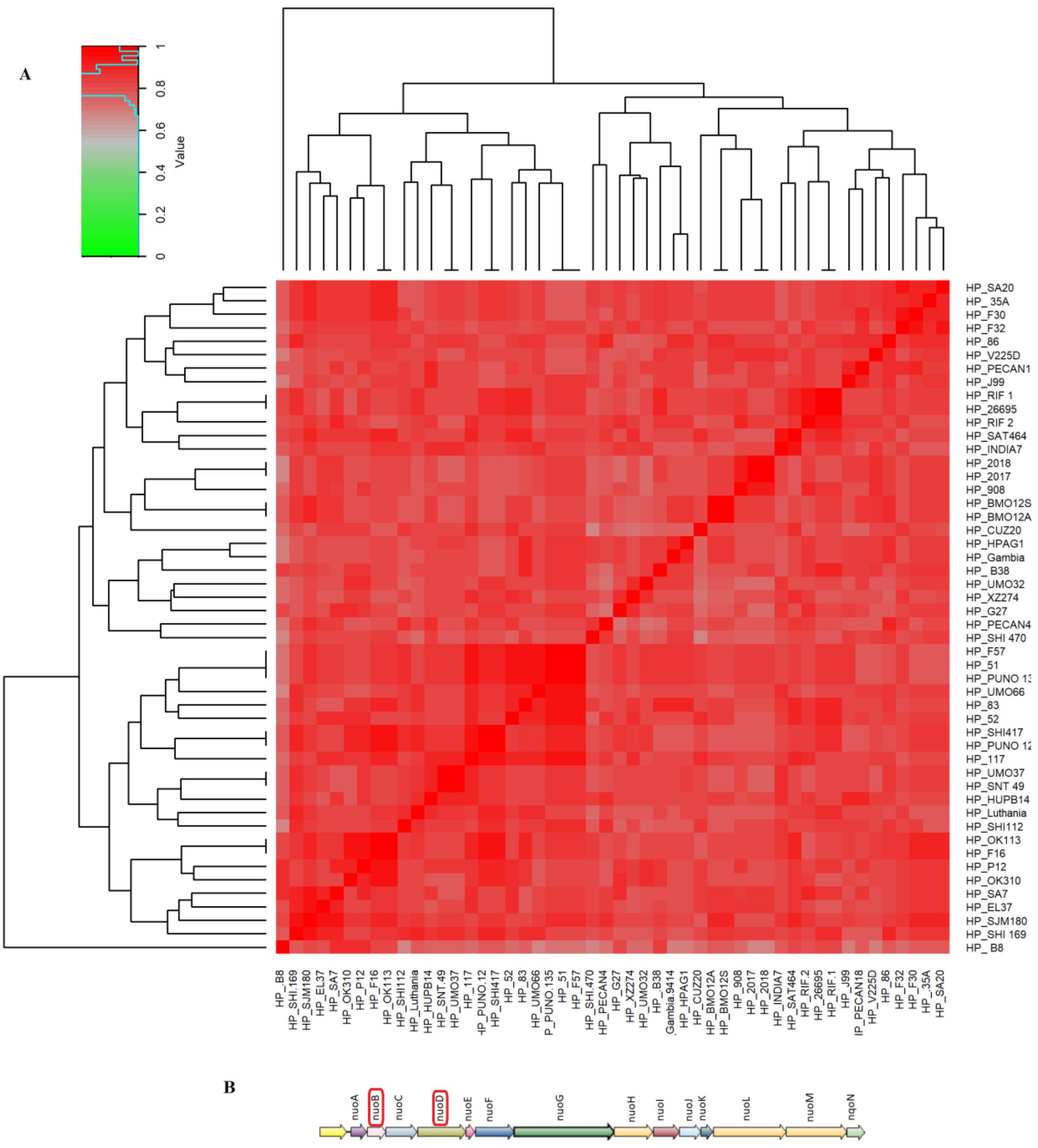

Supplementary Figure1. Comparative genomic study of Core_3 in fifty strains of *Helicobacter pylori*. A. Heatmap of Core_3 generated from square matrices of similarity score among the fifty *H. pylori* strains. B. Synteny of Core_3 Genomic island in all fifty *H. pylori* strains. Four missense mutations in the NuoB and NuoD genes have resulted in the emergence of benzimidazole-resistant *H. pylori* strains.

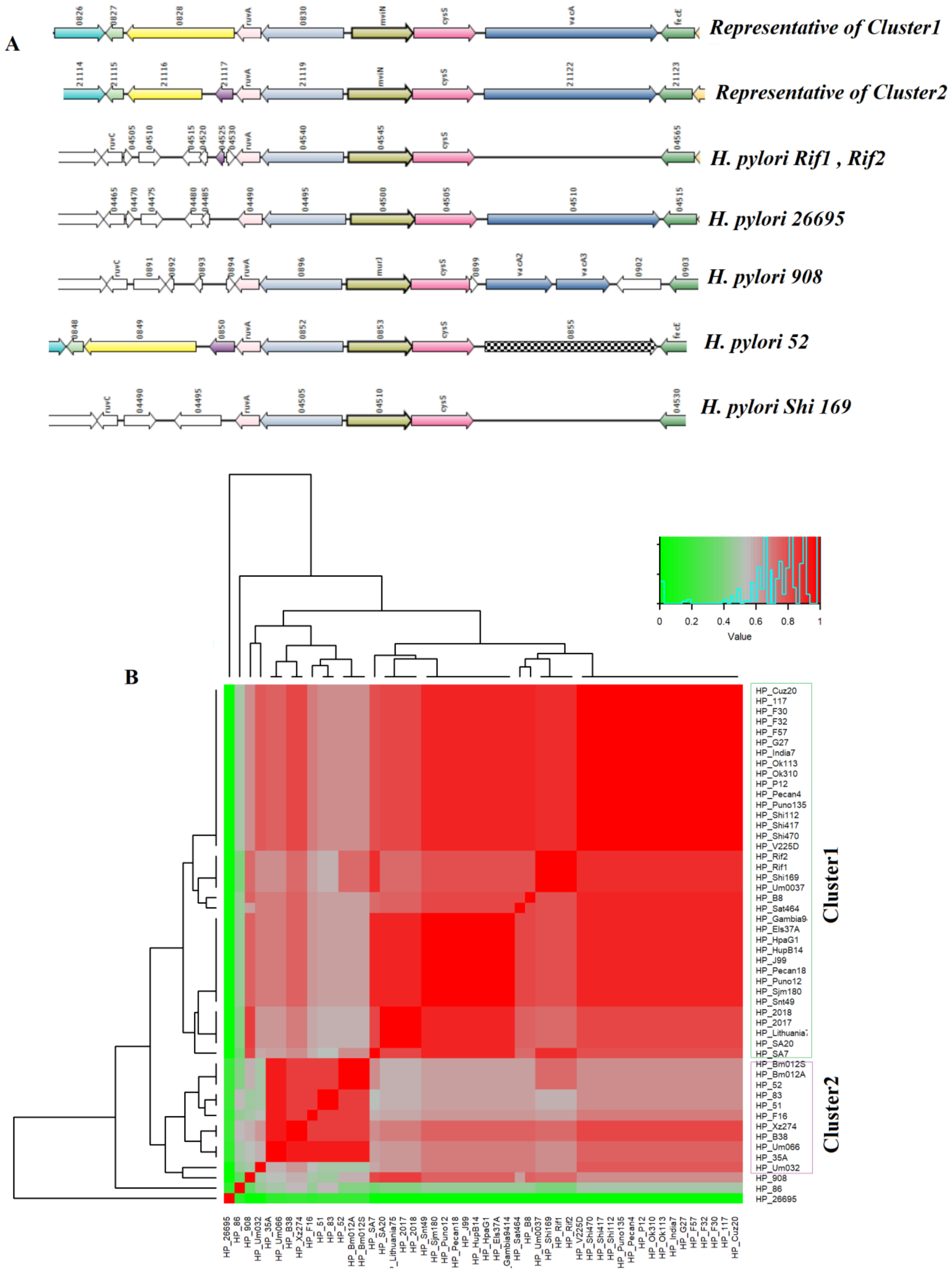

Supplementary Figure 2. Comparative genome study of Core_4. **A.** Synteny of representative strains of Core_4. **B.** Heatmap of similarity scores using hierarchical clustering. Fifty strains showed two major clusters. Red: S_m ≥0.70; Grey: S_m: ≥0.4-0.69, Green: S_m ≤0.39.

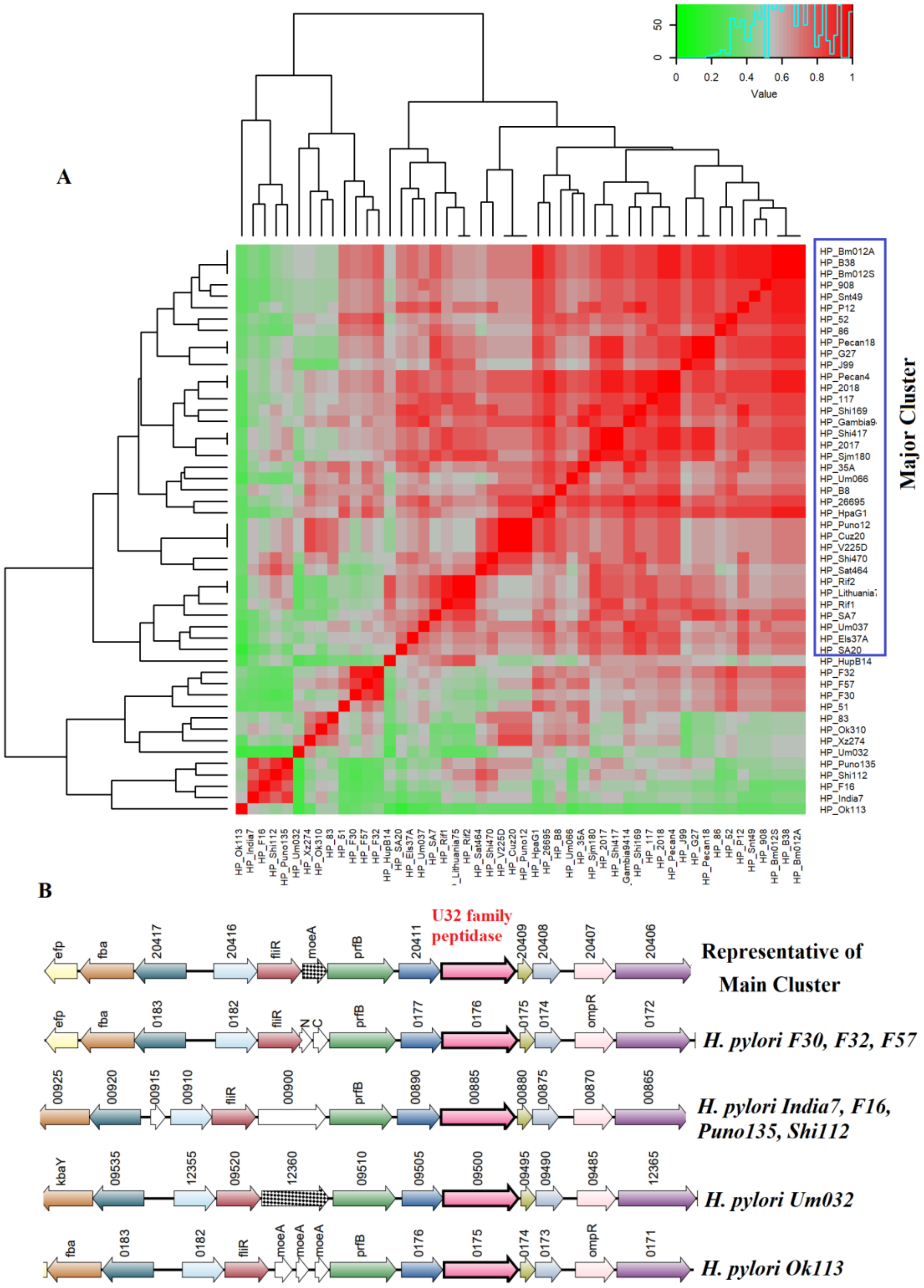

Supplementary Figure 3. Comparative genomic study of Core_6 in fifty strains of *Helicobacter pylori*. A. Heatmap of similarity scores using hierarchical clustering. Fifty strains showed one main cluster. Red: S_m ≥0.70; Grey: S_m: ≥0.4-0.69, Green: S_m ≤0.39. B. Synteny of representative strains of Core_6. U32 family protein was conserved among the strains.

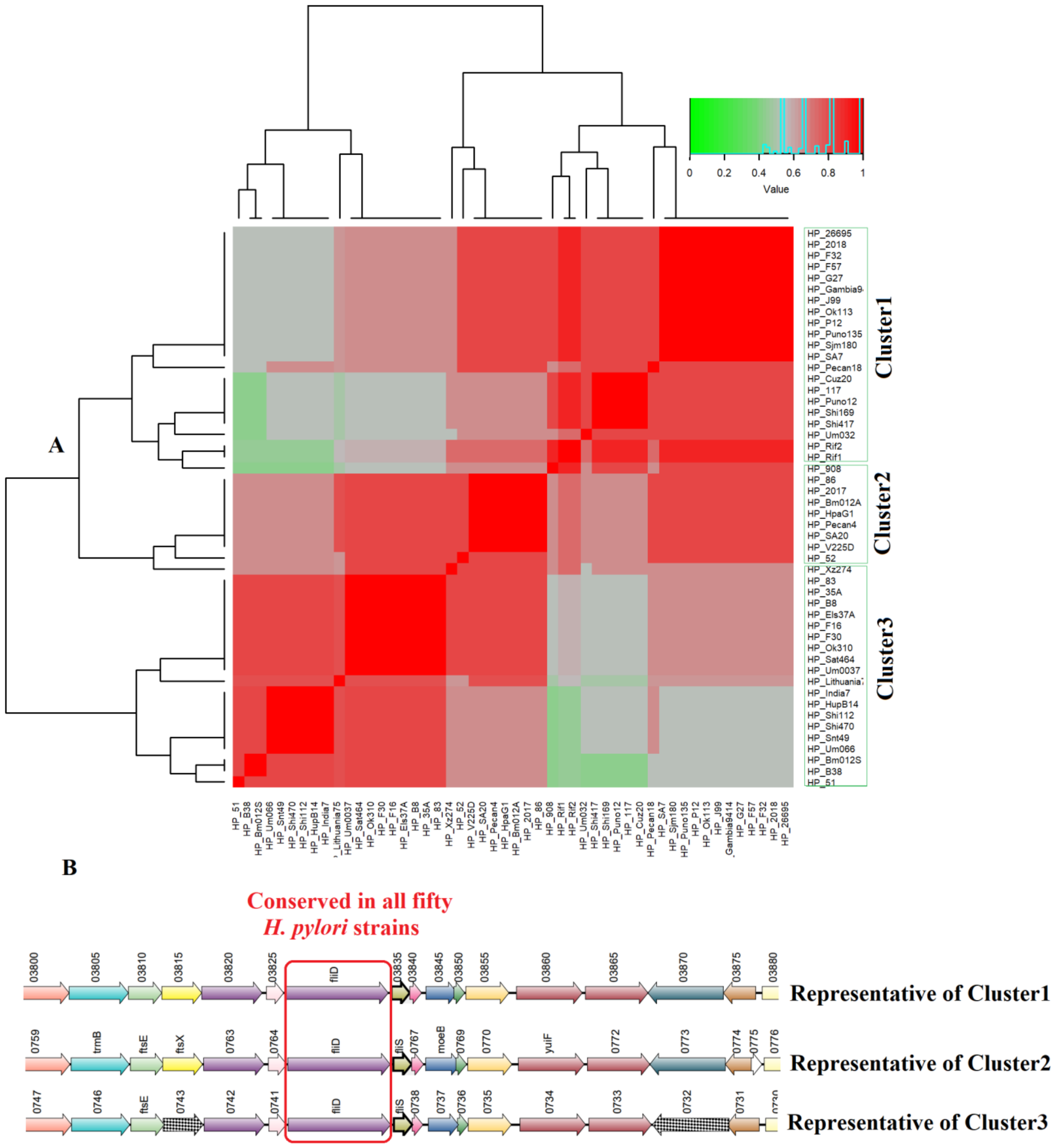

Supplementary Figure 4. Comparative genomic study of Core_7 in fifty strains of *Helicobacter pylori*. A. Heatmap of similarity scores using hierarchical clustering. Fifty strains formed three major clusters. Red: S_m ≥0.70; Grey: S_m: ≥0.4-0.69, Green: S_m ≤0.39. Fifty strains showed two main clusters. B. Synteny of representative strains from each clusters. *fliD* is present in all the fifty strains.

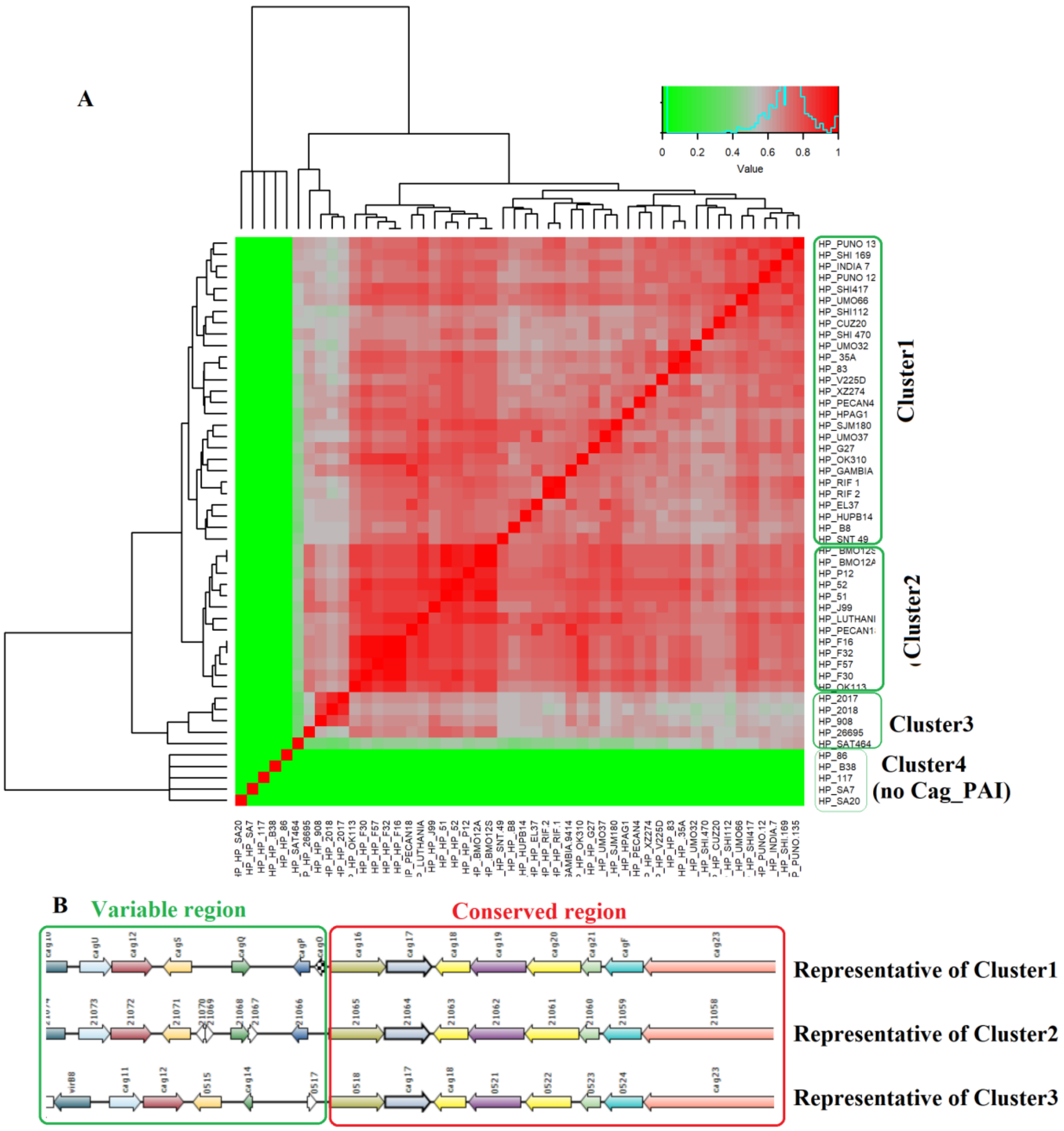

Supplementary Figure 5. Comparative genomic study of Cag Pathogenicity Island (Acc_25) in fifty strains of *Helicobacter pylori*. **A.** Heatmap of similarity scores using hierarchical clustering. Red: S_m ≥0.70; Grey: S_m: ≥0.4-0.69, Green: S_m ≤0.39. Fifty strains showed four main clusters. Cluster4 represents cag –ve strains. **B.** Synteny of representative strains from each cluster. The region highlighted in red is the most variable part of the Cag PAI.

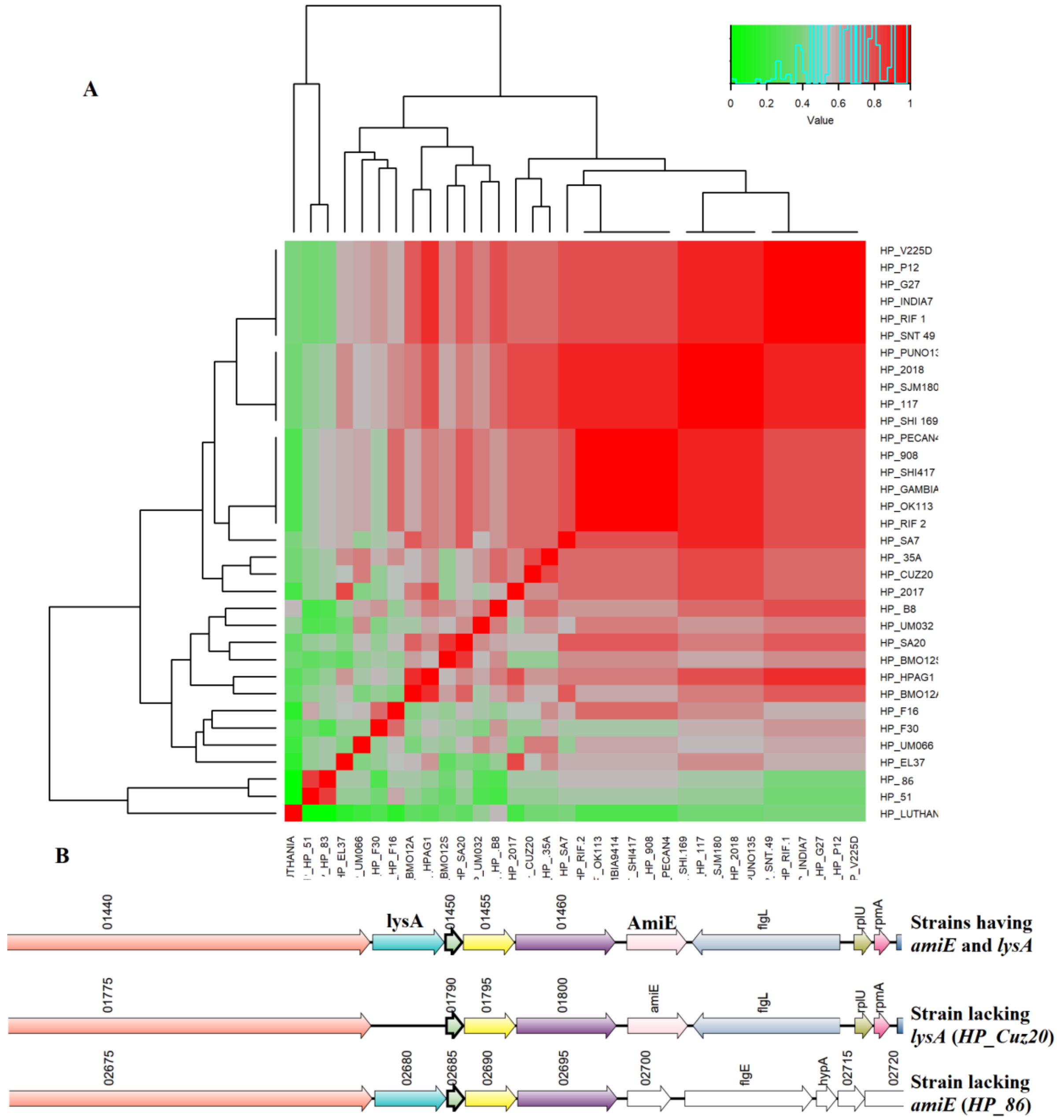

Supplementary Figure 6. Comparative genomic study of Acc_14 in fifty strains of *Helicobacter pylori*. **A.** Heatmap of similarity scores using hierarchical clustering. Red: S_m ≥0.70; Grey: S_m: ≥0.4-0.69, Green: Sm ≤0.39. **B.** Synteny of representative strains based on the presence and absence of *lysA* and *amiE* gene.

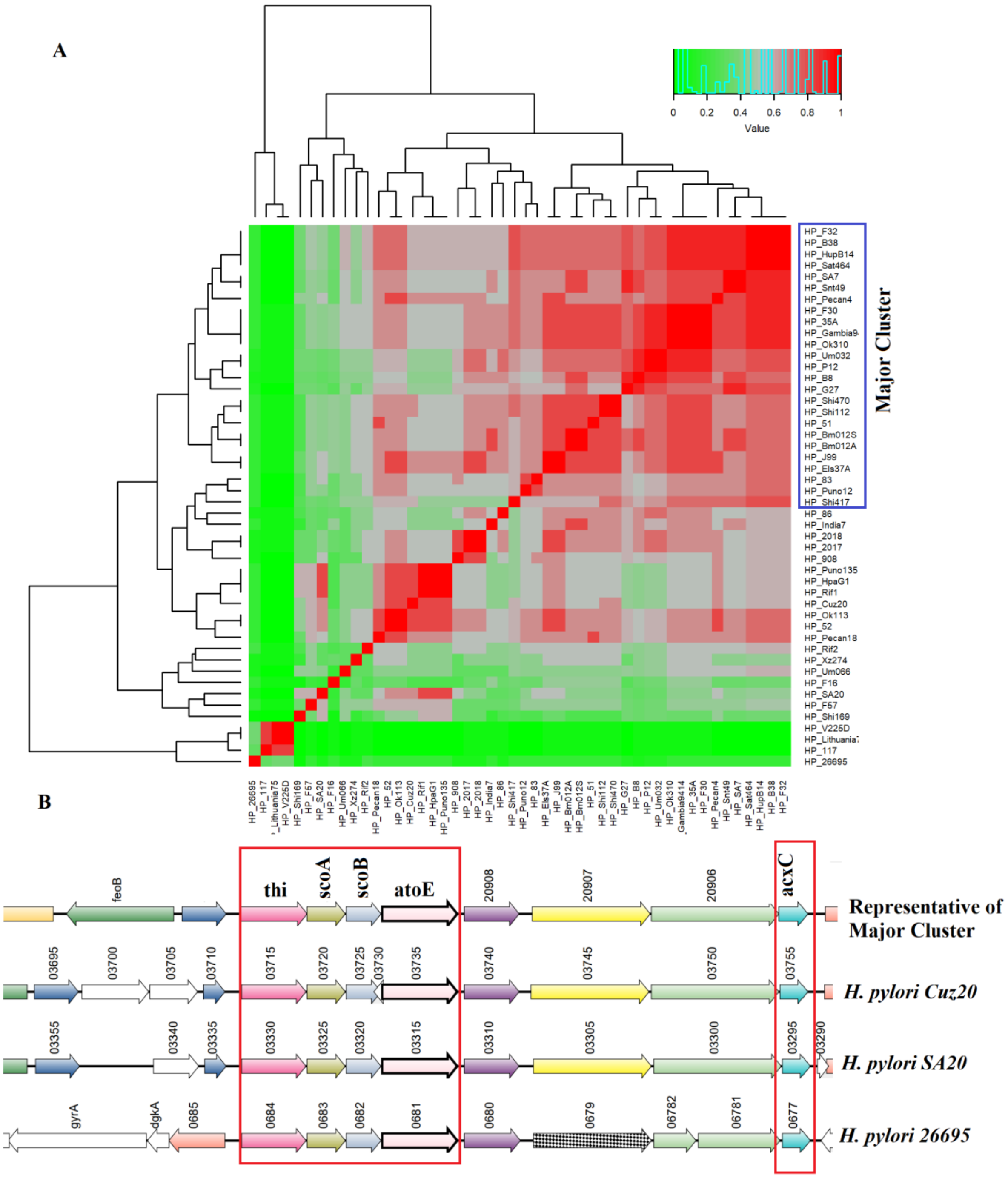

Supplementary Figure 7. Comparative genomic study of Acc_20 in fifty strains of *Helicobacter pylori*. **A.** Heatmap of similarity scores using hierarchical clustering. Fifty strains showed one main cluster. : S_m ≥0.70; Grey: S_m: ≥0.4-0.69, Green: S_m ≤0.39. **B.** Synteny of representative strains from each cluster. The region highlighted in red is the most conserved part.

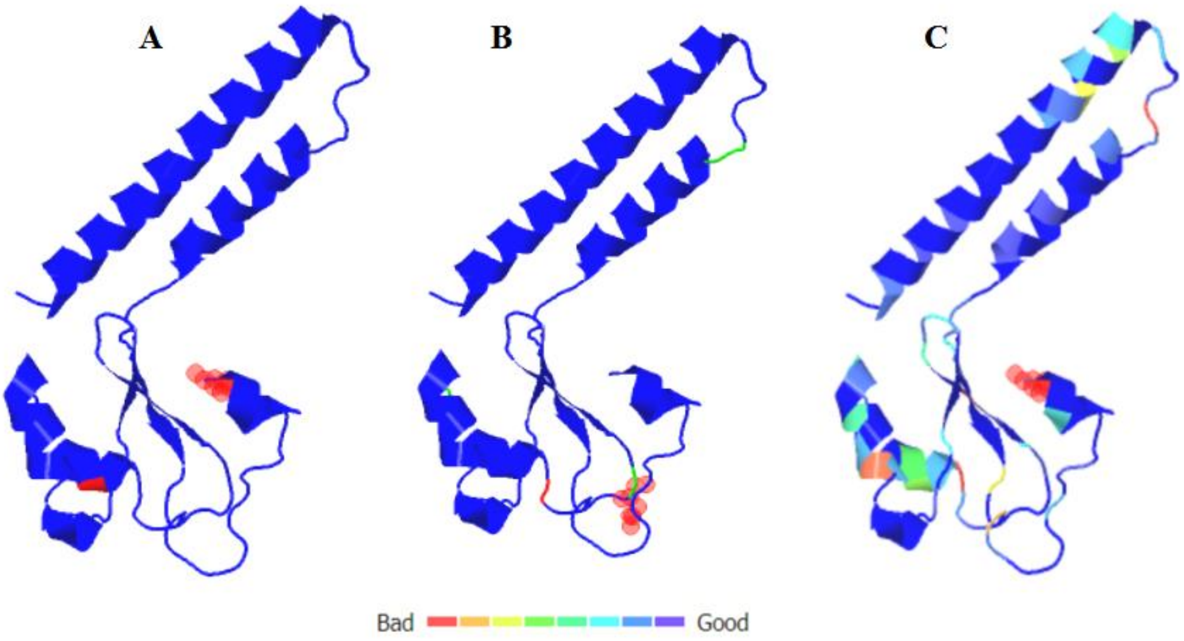

Supplementary Figure 8. Quality assessment of homology model of putative toxin. A. Rotamer analyis: Some side chains in the model may not have been modelled ideally. These are coloured in red and may indicate a problem with the backbone or underlying alignment in this region. B. Ramachandran analysis: Some residues in the model may lie in favorable (blue), allowed (green) or disallowed (red) regions of the Ramachandran plot. This colouring indicates residues that may have problems with the backbone phi/psi angles. C. Clash Analysis: Some atoms in some residues may lie too close to one another in the model. Residues are colored by how many clashes are observed. A large number of clashes could mean bad side chain placement.

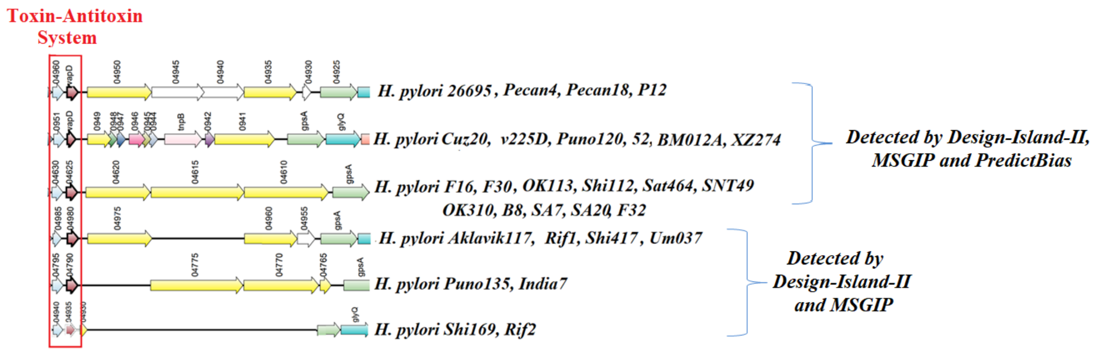

Supplementary Figure 9: Synteny study of the Acc_33. The representative synteny of eight *H. pylori* strains which were not identified by PredictBias is highlighted in the figure.

Supplementary Table 1: Disease association of 50 *H. pylori* strains

**Disease**	**Number of strains**
Duodenal Ulcer (DU)	8
Gastric Cancer (GC)	6
Gastric Ulcer (GU)	3
Gastritis	7
Peptic Ulcer (PU)	3
Unknown	23

Supplementary Table 2: Geographical Distribution of **50 *H. pylori* strains**

Country	Number of strains
**Canada**	**2**
**Malaysia**	**3**
**UK**	**1**
**Sweden**	**1**
**Spain**	**1**
**Lithuania**	**1**
**Italy**	**1**
**Germany**	**1**
**France**	**2**
**Korea**	**2**
**Japan**	**8**
**China**	**1**
**Australia**	**2**
**India**	**2**
**Venezuela**	**1**
**Peru**	**14**
**West and South African Countries**	**7**

Supplementary Table 3: Functional Characterization of thirty eight accessory islands identified by comparative study fifty *H. pylori* strains

Name	Function	Name	Function
Core_1	Ribosomal Protein	Acc_14	Lysine biosynthetic process
Core_2	Ribosomal Protein	Acc_15	Miscellaneous
Core_3	Antibiotic Resistance Island	Acc_16	Miscellaneous
Core_4	VacA island	Acc_17	Lipopolysaccharide biosynthesis
Core_5	Niche adaptation	Acc_18	Miscellaneous
Core_6	Promoting Ulceration	Acc_19	Miscellaneous
Core_7	Biofilm Formation	Acc_20	Metabolism
Acc_1	Niche adaptation	Acc_21	Miscellaneous
Acc_2	Miscellaneous	Acc_22	Miscellaneous
Acc_3	Miscellaneous	Acc_23	Membrane efflux protein
Acc_4	Bacterial Transport System	Acc_24	Miscellaneous
Acc_5	Biosynthesis	Acc_25	Cag pathogenicity Island
Acc_6	Miscellaneous	Acc_26	Miscellaneous
Acc_7	Miscellaneous	Acc_27	Metabolism
Acc_8	Miscellaneous	Acc_28	Terpenoid-quinone biosynthesis
Acc_9	Miscellaneous	Acc_29	Cell cycle
Acc_10	Miscellaneous	Acc_30	Metabolism
Acc_11	Miscellaneous	**Acc_31**	**Putative toxin**
Acc_12	Miscellaneous	Acc_32	Purine biosynthesis
Acc_13	Miscellaneous	**Acc_33**	**Toxin- antitoxin system**
Acc_34		Miscellaneous	

Supplementary Table 4: Number of *H. pylori* strains detected by different GI prediction tools

GI Names	***Design-Island-II***	MSGIP	PredictBias	ZislandExplorer	IslandPath-DIMOB	IslandPick
Acc_1	32	0	0	0	0	0
Acc_2	26	26	26	0	0	0
Acc_3	34	26	31	25	0	0
Acc_4	49	0	0	0	0	0
Acc_5	26	0	0	0	0	0
Acc_6	27	27	27	27	0	0
Acc_7	26	16	26	0	0	0
Acc_8	39	34	37	31	0	0
Acc_9	45	0	0	0	0	0
Acc_10	46	46	0	0	0	0
Acc_11	45	40	38	0	0	0
Acc_12	33	31	29	35	0	0
Acc_13	32	32	19	36	0	0
Acc_14	33	0	0	0	0	0
Acc_15	39	32	32	36	0	0
Acc_16	27	22	26	24	0	0
Acc_17	41	0	0	0	0	0
Acc_18	39	32	33	0	0	0
Acc_19	44	42	37	35	0	0
Acc_20	48	0	0	0	0	0
Acc_21	47	43	42	40	0	0
Acc_22	32	34	0	0	0	0
Acc_23	41	41	34	0	0	0
Acc_24	44	42	0	0	0	0
Acc_25	45	45	45	45	45	45
Acc_26	45	0	0	0	0	0
Acc_27	40	0	0	0	0	0
Acc_28	43	0	0	0	0	0
Acc_29	30	30	26	26	0	0
Acc_30	30	30	27	24	0	0
**Acc_31**	**5**	**5**	**5**	**5**	0	0
Acc_32	35	35	0	0	0	0
**Acc_33**	**29**	**29**	**21**	0	0	0
Acc_34	10	0	0	0	0	0
